# The *Drosophila* brain on cocaine at single-cell resolution

**DOI:** 10.1101/gr.268037.120

**Published:** 2021-10

**Authors:** Brandon M. Baker, Sneha S. Mokashi, Vijay Shankar, Jeffrey S. Hatfield, Rachel C. Hannah, Trudy F.C. Mackay, Robert R.H. Anholt

**Affiliations:** Center for Human Genetics, Department of Genetics and Biochemistry, Clemson University, Greenwood, South Carolina 29646, USA

## Abstract

Whereas the neurological effects of cocaine have been well documented, effects of acute cocaine consumption on genome-wide gene expression across the brain remain largely unexplored. This question cannot be readily addressed in humans but can be approached using the *Drosophila melanogaster* model, where gene expression in the entire brain can be surveyed at once. Flies exposed to cocaine show impaired locomotor activity, including climbing behavior and startle response (a measure of sensorimotor integration), and increased incidence of seizures and compulsive grooming. To identify specific cell populations that respond to acute cocaine exposure, we analyzed single-cell transcriptional responses in duplicate samples of flies that consumed fixed amounts of sucrose or sucrose supplemented with cocaine, in both sexes. Unsupervised clustering of the transcriptional profiles of a total of 86,224 cells yielded 36 distinct clusters. Annotation of clusters based on gene markers revealed that all major cell types (neuronal and glial) as well as neurotransmitter types from most brain regions were represented. The brain transcriptional responses to cocaine showed profound sexual dimorphism and were considerably more pronounced in males than females. Differential expression analysis within individual clusters indicated cluster-specific responses to cocaine. Clusters corresponding to Kenyon cells of the mushroom bodies and glia showed especially large transcriptional responses following cocaine exposure. Cluster specific coexpression networks and global interaction networks revealed a diverse array of cellular processes affected by acute cocaine exposure. These results provide an atlas of sexually dimorphic cocaine-modulated gene expression in a model brain.

Cocaine use presents a significant socioeconomic health problem (Kariisa et al. 2019; Substance Abuse and Mental Health Services Administration 2019). Although cocaine use results in arousal and euphoria, side effects include accelerated heart rate, mood swings, difficulty sleeping, loss of appetite, and cognitive distortions. Escalated consumption of cocaine can result in psychosis, cardiovascular disease, and stroke.

The propensity for cocaine use depends on genetic and environmental factors. Whereas much is known about the neurological effects of cocaine, information about genetic variants that are associated with variation in individual susceptibility to psychostimulant use remains incomplete. Furthermore, little is known about acute effects of cocaine consumption on genome-wide gene expression across the brain.

*Drosophila melanogaster* presents an advantageous model system for systems genetic analyses of cocaine consumption (Kaun et al. 2012). Flies can be reared rapidly in large numbers at low cost in defined genetic backgrounds and under controlled environmental conditions, and about 75% of disease-causing genes in humans have fly orthologs (Pandey and Nichols 2011). The crystal structure of the *Drosophila* dopamine transporter has been obtained and its binding site can accommodate cocaine (Wang et al. 2015). Exposure of cocaine elicits motor responses that resemble behaviors observed in rodents, and flies develop sensitization to repeated intermittent exposure to cocaine (McClung and Hirsh 1998; Filošević et al. 2018). Dopamine (Bainton et al. 2000), the dopamine transporter (DAT) (Wu and Gu 2003) and the serotonin transporter (SerT) (Corey et al. 1994; Demchyshyn et al. 1994; Borue et al. 2009, 2010) have been implicated in mediating cocaine-induced behaviors in flies (Li et al. 2000; Simon et al. 2009). Consistent with the actions of these neurotransmitters, overexpression of the vesicular monoamine transporter in both dopaminergic and serotonergic neurons decreases the response to cocaine (Chang et al. 2006). Thus, fundamental neural mechanisms affected by exposure to psychostimulants are conserved across phyla, from flies to humans.

Studies on inbred wild-derived, fully sequenced lines of the *Drosophila melanogaster* Genetic Reference Panel (Mackay et al. 2012; Huang et al. 2014) identified candidate genes associated with variation in consumption and development of preference for cocaine and methamphetamine (Highfill et al. 2019). Targeted RNA interference (RNAi) of gene expression implicated dopaminergic neurons and the mushroom bodies, central brain structures associated with experience-dependent modification of behavior, with consumption and development of preference for these psychostimulants (Highfill et al. 2019). However, RNAi-mediated reduction in expression of candidate genes in glia also affected cocaine consumption, suggesting that widespread brain regions contribute to cocaine-associated behavioral phenotypes. The present study aims to delineate the effects of acute cocaine consumption on genome-wide gene expression across the *Drosophila* brain.

## Results

### Cocaine consumption causes behavioral impairments

**Figure 1.**
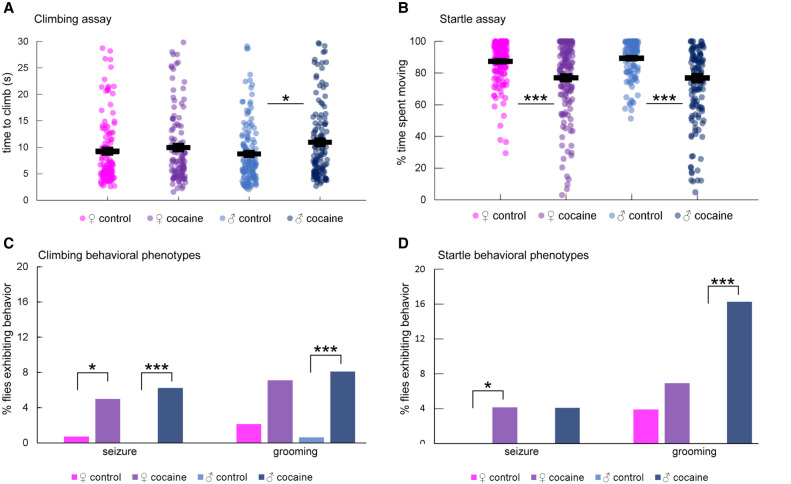
Behavioral characterization of Canton S (B) flies after cocaine exposure. (*A*) Negative geotaxis. The 7.5-cm climb time for each fly was measured. *n* = 120 (♀control), 114 (♀cocaine), 141 (♂control), 128 (♂cocaine). Horizontal lines represent means with standard error. Male flies exposed to cocaine took longer on average to climb compared to controls. (*) *P* = 0.0042; two-tailed Student's *t*-test. (*B*) Startle response. The percent time out of 45 sec that each fly spent moving following a 42-cm drop was measured. *n* = 155 (♀control), 145 (♀cocaine), 120 (♂control), 123 (♂cocaine). Horizontal lines represent means with standard error. Flies exposed to cocaine spent less time moving on average than controls. For females, (***) *P* = 4.68 × 10^−6^; for males, (***) *P* = 7.62 × 10^−13^; two-tailed Student's *t*-test. (*C*) Seizures and grooming activity during negative geotaxis. The percent of flies that exhibited seizures or grooming activity during the negative geotaxis assay after exposure to cocaine was measured. *n* = 142 (♀control), 141 (♀cocaine), 166 (♂control), 161 (♂cocaine). Both females and males exposed to cocaine exhibited seizure activity more than controls. For females, (*) *P* = 0.0361; for males seizure, (***) *P* = 0.0007; Fisher's exact test. Males exposed to cocaine also exhibited excessive grooming activity more than controls (grooming: [***] *P* = 0.0007; Fisher's exact test), but females did not show statistically significant differences. (*D*) Seizures and grooming activity during the startle response. The percent of flies that exhibited seizures or grooming activity during the startle assay after exposure to cocaine was measured. *n* = 155 (♀control), 145 (♀cocaine), 120 (♂control), 123 (♂cocaine). Female flies exposed to cocaine exhibited more seizure activity than controls ([*] *P* = 0.0121; Fisher's exact test), whereas male flies exposed to cocaine exhibited more grooming activity than control ([***] *P* = 0.00001; Fisher's exact test).

To assess the effects of acute cocaine exposure on fly behavior, we allowed males and females to ingest a fixed amount of sucrose or sucrose supplemented with cocaine within a maximal 2-h time period. We measured negative geotaxis, an innate locomotor behavior, to assess locomotion impairments, and startle behavior as a measure of sensorimotor integration (Fig. 1; Supplemental Table S1). Male flies exposed to cocaine took longer to climb in the negative geotaxis assay than control flies, whereas females appeared unaffected (Fig. 1A). Both male and female flies exposed to cocaine spent less time moving after being subjected to a mechanical disturbance (Fig. 1B; Supplemental Video S1). The average reduced locomotor activity in both assays might result from excessive grooming behavior in a fraction of male flies exposed to cocaine (Fig. 1C,D; Supplemental Video S2). In addition, we observed seizures in a small percentage of flies after cocaine intake during the negative geotaxis assay (Supplemental Video S3). Seizures rarely occurred in controls (Fig. 1C,D). Collectively, these experiments provide evidence that acute exposure to cocaine results in neurological impairments.

### Single-cell RNA-seq reveals cocaine-modulated gene expression in neurons and glia

**Figure 2.**
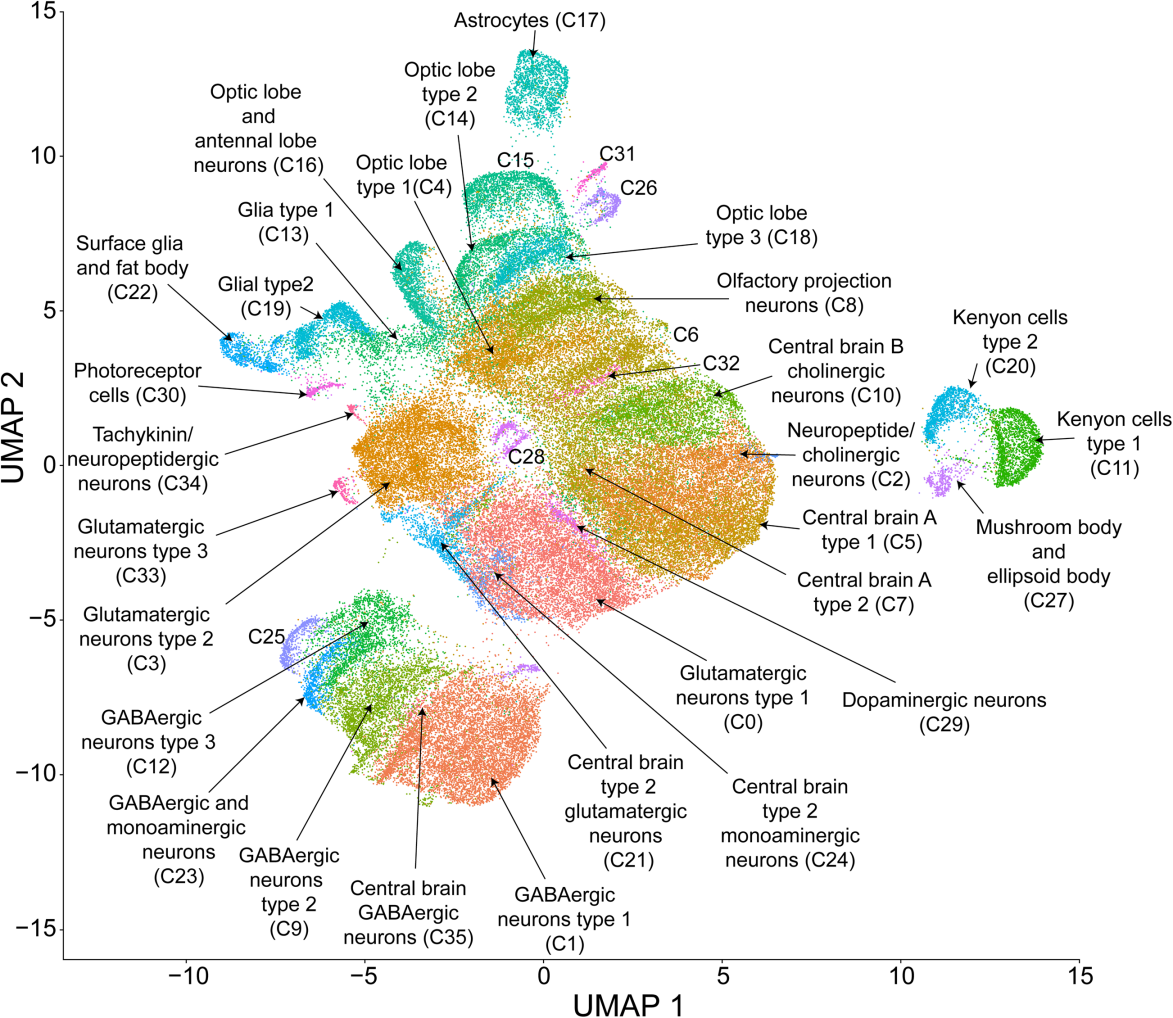
UMAP visualization and clustering of single-cell expression data. Cells were clustered based on their expression pattern using the unsupervised shared nearest neighbor (SNN) clustering algorithm. Individual dots represent each cell and the colors of the dots represent the cluster to which the cells belong. Identification of cell types from clusters was performed by cross-referencing cluster-defining genes across FlyBase (Thurmond et al. 2019) and published literature (see Supplemental Table S4).

To assess effects of cocaine consumption on brain gene expression, we analyzed single-cell transcriptional responses in duplicate samples of flies that consumed fixed amounts of sucrose or sucrose supplemented with cocaine in both males and females (Supplemental Table S2). Visualization of the resulting integrated data set using the Uniform Manifold Approximation and Projection (UMAP) nonlinear dimensionality reduction method (Becht et al. 2019) showed that no single cluster was dominated by a specific sample, sex, condition, or replicate and that there was considerable homogeneity (i.e., an even distribution of cells from samples) across the entire data set (Supplemental Fig. S1). We identified 691 differentially expressed genes in males and 322 in females following acute exposure to cocaine, of which ∼69% have human orthologs (Supplemental Table S3). Unsupervised clustering of the integrated data set based on the expression profiles of individual cells resulted in 36 distinct, stable clusters (Fig. 2). We assessed the stability of clustering by examining the relationship between the number of new clusters identified and the granularity resolution parameter (Butler et al. 2018). At a resolution of 0.8, the number of clusters stabilized and the resolution had to be increased significantly from this value in order to add new clusters, indicating that saturation in the diversity of expression profiles had been reached.

**Figure 3.**
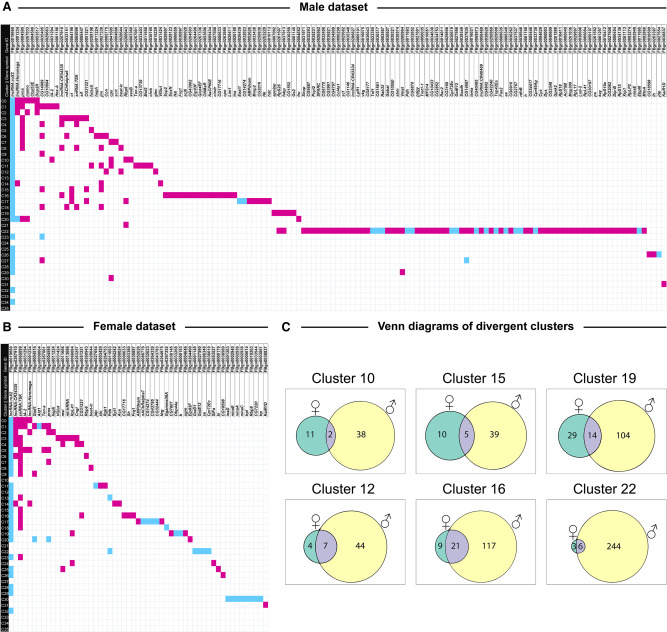
Distribution of differentially expressed genes across clusters in males (*A*) and females (*B*) exposed to cocaine, and Venn diagrams showing overlap between differentially expressed genes in males and females (*C*). To identify clusters with unique gene expression patterns following acute exposure to cocaine, we filtered the list of differentially expressed genes to only show the strongest responses (|log_e_FC| > 1.0, Bonferroni-adjusted *P*-value < 0.05) to construct an expression matrix. Differentially expressed genes are listed on the *top* (columns) and cell clusters are represented by the rows. Magenta boxes show up-regulation and turquoise boxes show down-regulation of gene expression as a result of exposure to cocaine. Panel *C* shows Venn diagrams of clusters with sexually dimorphic responses to cocaine exposure. The numbers within each Venn diagram represent the unique and shared differentially expressed (|log_e_FC| > 0.5, Bonferroni-adjusted *P*-value < 0.05) genes due to cocaine exposure from DGE analysis performed for the corresponding cluster in male and female data sets separately.

We identified cell types corresponding to each cluster using the top marker genes from each cluster, obtained by comparing each cluster's gene expression profile against the rest of the data set and filtered by |log_e_FC| > 0.5, Bonferroni-adjusted *P*-value < 0.05. Annotation of clusters based on their gene markers revealed that all major cell types (neuronal and glial) as well as neurotransmitter types from most brain regions, including the mushroom bodies, were represented (Fig. 2; Supplemental Table S4). Differential expression analysis within individual clusters indicated cluster-specific transcriptional responses to cocaine. Especially, clusters corresponding to glia and Kenyon cells of the mushroom bodies showed transcriptional responses following cocaine exposure (Fig. 3A,B). Thus, acute exposure to cocaine elicits rapid widespread changes in gene expression throughout the brain.

### Cocaine-modulated changes in gene expression are sexually dimorphic

We first analyzed differential expression by combining the male and female samples. There was a significantly greater number of genes up-regulated than down-regulated across all clusters in response to cocaine. Based on the number of strongly differentially expressed genes (|log_e_FC| > 1, Bonferroni-adjusted *P*-value < 0.05), clusters corresponding to surface glia (C22), unannotated cluster C16, astrocytes (C17), and Kenyon cells (C11) showed the largest responses to cocaine (Supplemental Fig. S2). In addition, a core set of genes, selected based on their ranks from the TopKLists consistency analysis (Schimek et al. 2015), show that they responded globally to cocaine exposure (Supplemental Table S5). These genes include: *Rpl41*, *IA-2*, and the long noncoding RNAs *CR34335* and *mt:lrRNA*, which were up-regulated; and *roX2* and *ninaE,* which were down-regulated after consumption of cocaine (Supplemental Fig. S2; Supplemental Tables S5, S6).

Examination of males and females separately revealed extensive sexual dimorphism in the response to cocaine. Consistent with effects on organismal phenotypes (Fig. 1), males showed more widespread changes in cocaine-modulated transcript abundances than females (Fig. 3A,B), When we consider only highly differentially expressed genes (|log_e_FC| > 1.0, Bonferroni-adjusted *P*-value < 0.05), we can construct expression matrices of 133 genes in males (Fig. 3A) and 54 genes in females (Fig. 3B). Clusters C11 (Kenyon cells), C16 (not annotated), C17 (astrocytes), and C22 (surface glia) had unique responses compared to the rest of the clusters in both males and females; C22 shows the most extensive cocaine-induced changes in transcript abundances in males (Fig. 3A). In addition to differences in the magnitude of cocaine-modulated gene expression between the sexes, we also observe differences in direction, in which up-regulation in one sex corresponds to down-regulation in the other. Overall, there was little overlap between the sexes—especially in clusters C10, C12, C15, C16, C19, and C22 (Fig. 3C; Supplemental Tables S7–S9). Thus, although cocaine-modulated changes in gene expression are widespread throughout the brain in both sexes, specific changes in transcript abundances are distinct between males and females.

### Coexpression networks highlight effects of cocaine on diverse cellular processes

Reactome analysis of pathway enrichment (Fabregat et al. 2018) in C11 and C20 in females, which, based on biomarkers, represent Kenyon cells of the mushroom bodies, highlighted inositol phosphate metabolism (Supplemental Table S10), suggesting a role for cocaine in modulating signal transduction. We were unable to assign a specific identity to C16, which might be comprised of a mixture of neurons from the antennal lobe and optic lobe (Supplemental Table S4). However, pathway analysis of male C16 revealed enrichment of multiple signal transduction pathways, including pathways related to G protein-coupled receptor signaling, activation of serotonin and AMPA- and NMDA-type glutamate receptors, activation of axonal growth inhibition, and Class A/1 Rhodopsin-like receptor signaling (Supplemental Table S10).

In contrast to the signal transduction elements associated with the neuronal C11 and C16 clusters, different cellular mechanisms are associated with cocaine exposure in C17 and C22, which represent astrocytes and surface glia, which comprise the blood-brain barrier in the fly, respectively (Supplemental Table S10). Functional enrichment analysis yielded few differentially expressed genes for females, but for C22 in the male data set, it revealed enrichment of Notch activation and signaling, degradation of GABA, immune pathways related to NF-kB activation, cytokine production and Toll-like receptor signaling, and nonsense-mediated decay and translation initiation (Supplemental Table S10). These observations are in line with expected functions of glia (Kremer et al. 2017).

**Figure 4.**
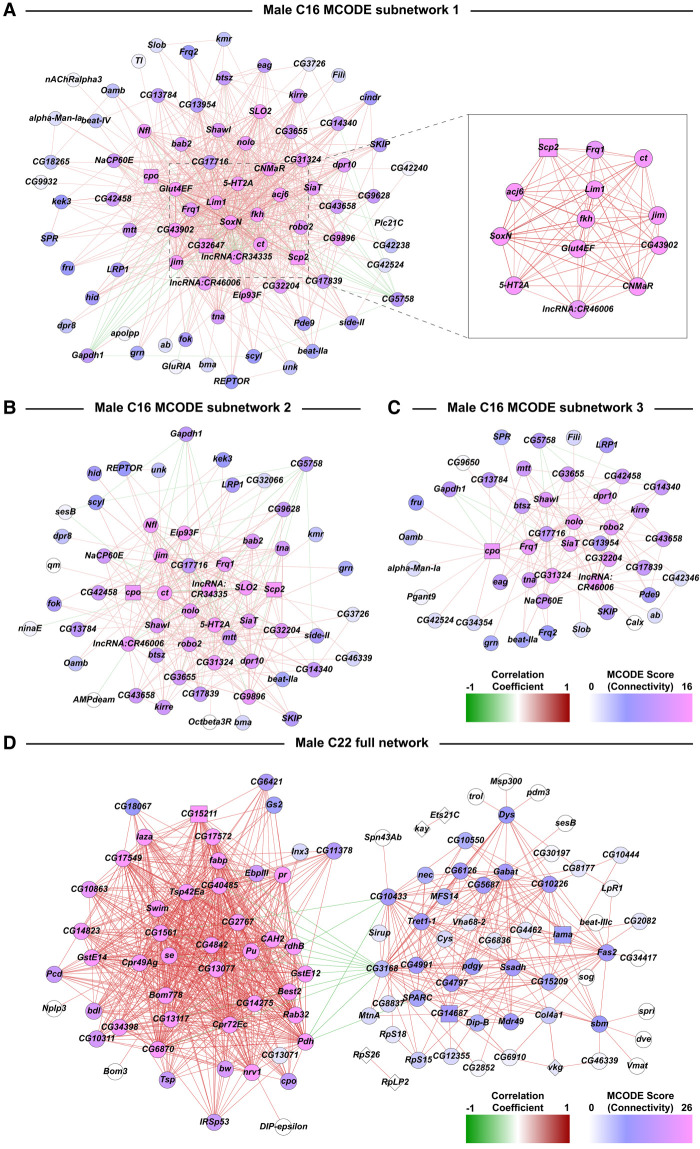
Subnetworks from coexpression network analyses of DEGs from the male C16 and C22 clusters. Networks are constructed from Pearson coefficient-based coexpression values calculated from scaled data of genes that were differentially expressed (filtered for |log_e_FC| > 0.5, Bonferroni-adjusted *P*-value < 0.05) due to cocaine exposure. Coexpressions have been filtered using Random Matrix Theory. (*A*–*C*) MCODE subnetworks derived from the full network of male cluster C16. The inset in *A* corresponds to a subset of genes within the subnetwork that have very strong correlation coefficient values with each other compared to the rest of the data set. Colors of the dots represent the connectivity index derived from MCODE scores. Colors of edges represent the positive (red) and negative (green) correlations. (*D*) Coexpression network analysis of DEGs from the male C22 cluster.

We used Random Matrix Theory (RMT) (Gibson et al. 2013) to construct coexpression networks of cocaine-modulated differentially coexpressed genes for selected clusters with enough differentially expressed input genes. Across all cell clusters, we find genes of unknown function and genes encoding long noncoding RNAs, which are likely to play a regulatory role (Everett et al. 2020). We present examples of coexpression networks for C16 males (Fig. 4A–C; Supplemental Fig. S3) and C22 males (Fig. 4D; Supplemental Fig. S4).

Coexpression analysis for C16 reveals a highly interconnected network that could be partitioned into three subnetworks using Molecular Complex Detection (MCODE) stratification (Bader and Hogue 2003). Central genes include transcriptional regulators associated with development, including dendrite morphogenesis (*Lim1*, *jim*) as well as signal transduction (*5-HT2A, CNMaR*) (Fig. 4A; Supplemental Fig. S3). Random Matrix Theory identified two major subnetworks within the interaction network that represents C22 (Fig. 4D; Supplemental Fig. S4). The two subnetworks in C22 were connected by only three genes (*CG3168*, *CG10433*, and *CG15209*) through negative correlation (Fig. 4D; Supplemental Fig. S4). Of the three genes, *CG3168*, which belongs to the SLC22 family of organic ion transporters, had the largest number of negative correlations linking the two large subnetworks. This gene is expressed in the blood-brain barrier of flies and postulated to play a role in chemoprotection of the brain (Hindle and Bainton 2014). Further stratification using the MCODE algorithm resulted in three tightly clustered C22 subnetworks. The C22 male interaction network comprises genes associated with oxidation-reduction (*se, Ssadh*) and redox reactions, particularly the glutathione system (*GstE12*, *GstE14, se*), as well as cell adhesion (*SPARC, bdl, Tsp, Fas2*).

**Figure 5.**
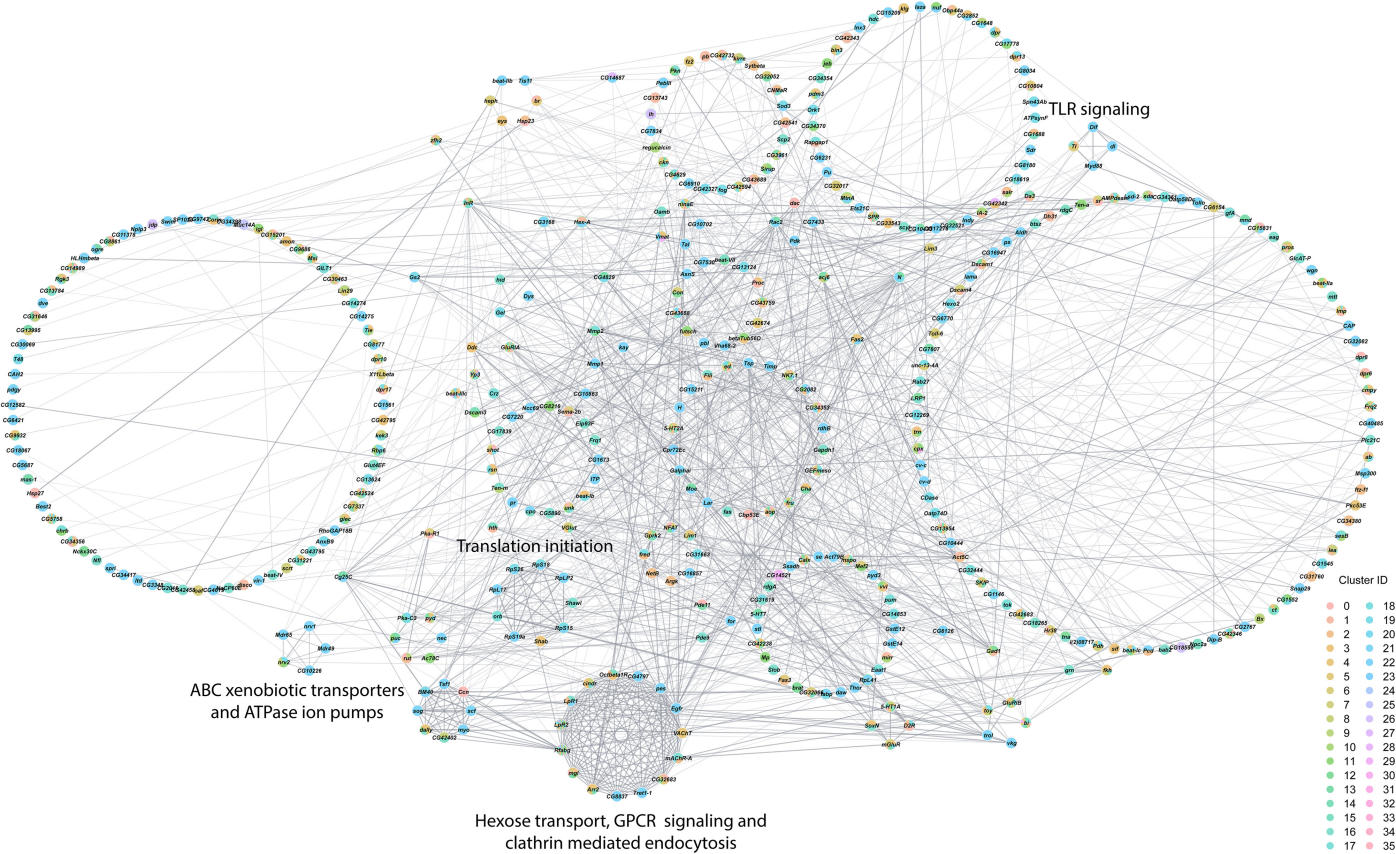
Interaction network analysis of DEGs from all clusters in the male data set. Network constructed from interactions calculated using StringApp plugin within Cytoscape for genes that were differentially expressed (filtered for |log_e_FC| > 0.1 and Bonferroni-adjusted *P*-value < 0.05) in all clusters from the male data set. Gray edges represent interactions. Genes that were differentially expressed in multiple clusters are depicted as pie charts with each color representing the respective cluster. Genes are grouped into circles based on their MCODE connectivity scores. Annotations of these circular groups represent the pathways that are enriched for the genes within these groups. Bonferroni-adjusted *P*-value < 0.05 was considered as significant for enrichment in the statistical overrepresentation tests.

**Figure 6.**
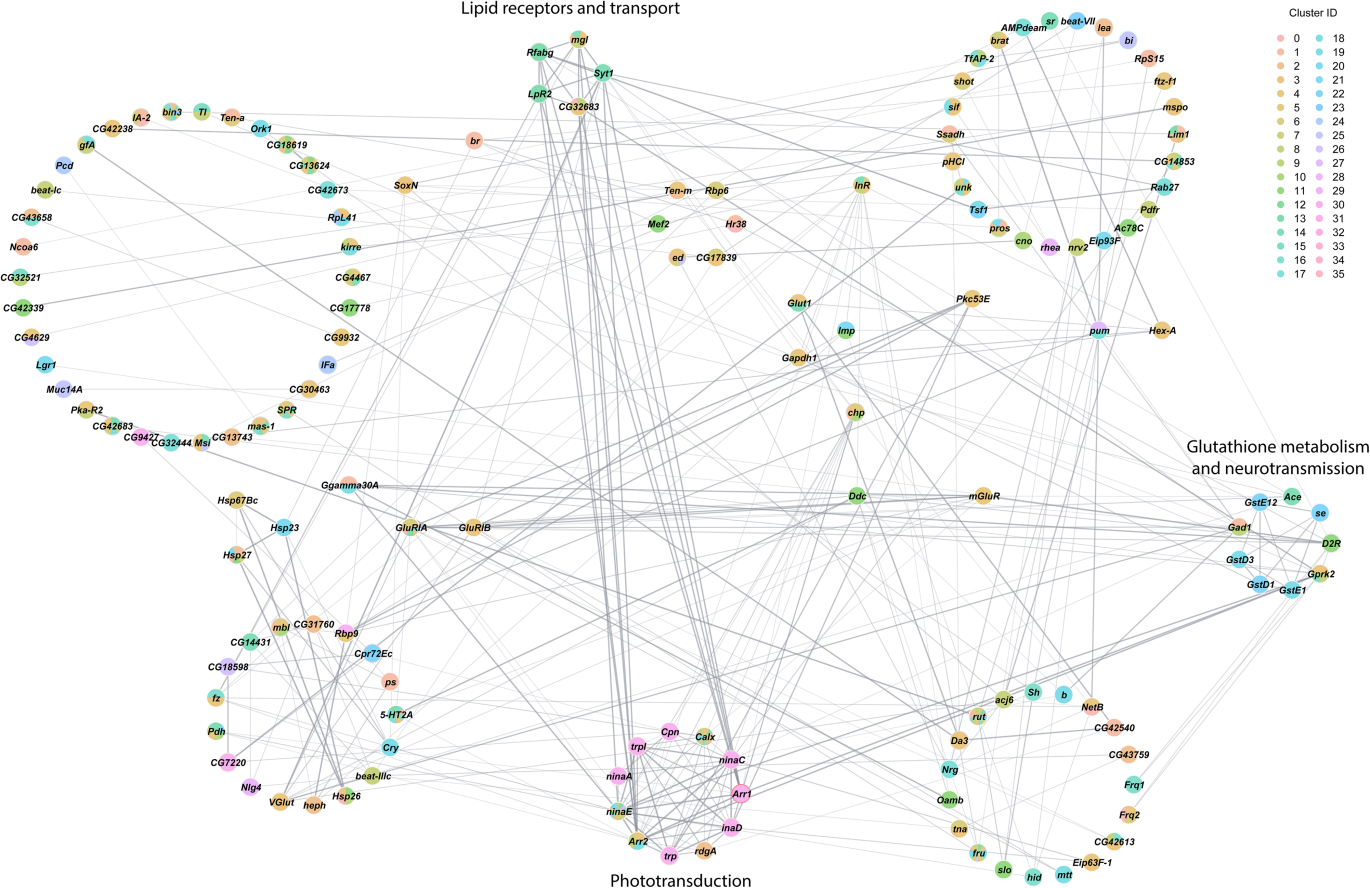
Interaction network analysis of DEGs from all clusters in the female data set. Network constructed from interactions calculated using StringApp plugin within Cytoscape for genes that were differentially expressed (filtered for |log_e_FC| > 0.1 and Bonferroni-adjusted *P*-value < 0.05) in all clusters from the female data set. Gray edges represent interactions. Genes that were differentially expressed in multiple clusters are depicted as pie charts with each color representing the respective cluster. Genes are grouped into circles based on their MCODE connectivity scores. Annotations of these circular groups represent the pathways that are enriched for the genes within these groups. BH-FDR adjusted *P*-value < 0.05 was considered as significant for enrichment in the statistical overrepresentation tests.

Finally, we assessed interaction networks among differentially expressed genes across all cell clusters separately for males and females (Figs. 5, 6). The global transcriptional response to cocaine in males is captured by a complex network of interconnected modules (Fig. 5). Functional analyses reveal modules associated with Toll-like receptor signaling, ABC xenobiotic transporters and ATPase ion pumps, translation initiation, and hexose transport, G protein-coupled receptor signaling and clathrin-mediated endocytosis. The female network has fewer genes and contains modules associated with phototransduction, lipid receptors and transport, and glutathione metabolism and neurotransmission (Fig. 6). In each network, multiple cell clusters contribute to the organization of each network module, indicating that the transcriptional response to cocaine is coordinated not only within but also across different cells throughout the brain.

## Discussion

Unlike humans and rodent models of substance abuse, *Drosophila* enables comprehensive single-cell transcriptomics analyses of living cells across the entire brain in a single analysis (Davie et al. 2018). We generated an atlas of cocaine-modulated gene expression changes in the fly brain and found that transcriptional changes in response to acute consumption of cocaine are rapid, widespread in both neurons and glia, and sexually dimorphic. We performed the experiments in duplicate to establish cross-validation. Transcript abundance levels are influenced by circadian time (Claridge-Chang et al. 2001; Sivachenko et al. 2013; Krzeptowski et al. 2018). Therefore, we performed all experiments within a defined window of circadian time. Because the transcriptional profiles we obtained provide a single ‘snapshot’ in time, we cannot extract information about the temporal progression of the transcriptional response to cocaine to determine whether or to what extent transcriptional responses in different cell populations occur sequentially or in parallel. Also, we cannot draw inferences as to which changes in gene expression might lay a foundation for development of cocaine preference (Highfill et al. 2019), because we only assessed acute transcriptional responses following a single exposure to cocaine.

### The response to cocaine is sexually dimorphic

Previous studies on the *Drosophila melanogaster* Genetic Reference Panel (Mackay et al. 2012; Huang et al. 2014) have documented sexual dimorphism in the genetic architectures of a wide range of morphological (Zwarts et al. 2015), life history (Nuzhdin et al. 1997; Huang et al. 2020), and behavioral traits (Shorter et al. 2015; Harbison et al. 2019), including cocaine consumption and preference (Highfill et al. 2019). However, sexual dimorphism in the transcriptional response following acute exposure to cocaine is extreme compared to any previous studies and is mirrored and consistent with the behavioral phenotypes (Fig. 1). It is possible that differences in cocaine metabolism between males and females may contribute to this sexual dimorphism. The sexual dimorphism we observe is in line with previous studies that show reduced locomotion and increased grooming in flies given low doses of cocaine, with males showing greater impairments in behavior (McClung and Hirsch 1998). We note, however, that changes in gene expression are not, a priori, necessary for cocaine to elicit behavioral effects.

### Cocaine-modulated gene expression in the mushroom bodies

Transcriptional effects of cocaine exposure are evident in all cell clusters, but among neuronal populations, the Kenyon cells of the mushroom bodies (represented by C11 and C20) have especially large responses to cocaine. The mushroom bodies are integrative centers associated with experience-dependent modulation of behavior and have previously been implicated in development of preference for cocaine intake (Highfill et al. 2019). Acute cocaine consumption leads to changes in gene products associated with signal transduction, including phosphatidyl inositol-mediated signaling in C11 in males (Supplemental Table S10), as well as cyclic AMP-mediated signaling, which is evident from increased expression of rutabaga (*rut*), in C20 in females (Supplemental Tables S8, S10). *rut* encodes a calcium-calmodulin-dependent adenylyl cyclase, implicated in learning and memory (Levin et al. 1992) and behavioral responses to ethanol (Rodan et al. 2002; Xu et al. 2012). *cpx*, which is involved in the functioning of the SNARE complex at the synapse (Scholz et al. 2019) and may affect neurotransmission, is up-regulated in males in response to cocaine. Kenyon cells receive dopaminergic input, and acute exposure to cocaine results in altered expression of *Ddc*, which encodes Dopa decarboxylase, and down-regulation of *Dop2R*, which encodes a dopamine receptor in C11 in females (Supplemental Table S8). *slo*, which regulates neurotransmitter release at synapses (Jepson et al. 2014), is up-regulated in response to cocaine, as is *Rgk1*, which plays a role in negative regulation of calcium channel activity (Murakami et al. 2017). Down-regulation of *jdp* can lead to dopamine blockade through its activity as a cochaperone in synaptic vesicle release (Ye et al. 2004). Based on the collective data, cocaine-induced modulation of gene expression appears to result in altered synaptic regulation in the mushroom bodies.

### Cocaine-modulated gene expression in glia

In addition to cocaine-modulated changes in gene expression in neurons, acute exposure to cocaine results in altered transcript abundances in different populations of glia (C13, C17, C19, C22), including surface glia (C22) and astrocytes (C17). Mutants of *moody*, which encodes two G protein-coupled receptor isoforms localized to surface glia, have increased sensitivity to cocaine (Bainton et al. 2005). The surface glia, represented by perineurial and subperineurial glia, act as the blood-brain barrier (DeSalvo et al. 2014; Kremer et al. 2017) and mediate the innate immune response (Kounatidis and Chtarbanova 2018). Genes associated with the blood-brain barrier in *Drosophila* are also up-regulated in response to cocaine. This includes *ogre,* which regulates gap junction channel activity (Holcroft et al. 2013; Spéder and Brand 2014), and *Nrg,* which plays a role in cell adhesion in the blood-brain barrier (Kanda et al. 2019), in females.

Acute exposure to cocaine causes changes in expression of genes involved with Toll-like receptor (TLR) signaling, nuclear factor kappa B (NF-kB) activation, cytokine production, and glutathione metabolism (Figs. 5, 6). TLR signaling has been associated with response to cocaine (Zhu et al. 2018), likely due to the interaction of cocaine with TLR on microglia (Northcutt et al. 2015), and cocaine activates the NF-kB pathway in the nucleus accumbens of mice (Russo et al. 2009; Muriach et al. 2010).

Astrocytes provide metabolic support for neurons (Tsacopoulos and Magistretti 1996) and regulate neuronal NMDA receptors and synaptic plasticity (Haydon et al. 2009). Glutamatergic neurons feature prominently in C1, C3, C21, and C33. Studies on rats have shown that cocaine is toxic to astrocytes and that loss of astrocytes leads to dysfunctional neuron-glia communication (Badisa et al. 2013, 2014, 2015). *Eaat1*, which is highly expressed in astrocytes (Supplemental Table S4), encodes a transmembrane glutamate transporter involved in glia-neuron communication (MacNamee et al. 2016). *Eaat1* is down-regulated in response to cocaine in males and has been associated with lifespan (Mazaud et al. 2019), long-term memory (Matsuno et al. 2019), seizures (Jen et al. 2005; Cho et al. 2018), and ataxia (Jen et al. 2005; Parinejad et al. 2016). Episodic ataxia due to a mutation in this gene (Parinejad et al. 2016) suggests that altered expression of *Eaat1* in astrocytes could play a role in cocaine-induced locomotor effects.

### Translating findings from the *Drosophila* model to cocaine sensitivity in humans

Like flies, people show sexually dimorphic effects of cocaine use. Although substance use disorders are more prevalent in males, females are more likely to escalate their drug usage to the compulsive stage faster than males (Haas and Peters 2000; Westermeyer and Boedicker 2000), report more negative effects during withdrawal, and have greater relapse than males (Becker and Koob 2016). Females metabolize cocaine faster than males, as is evident from lower levels of cocaine metabolites in the bloodstream of females compared to males who have consumed equal amounts of cocaine (Lukas et al. 1996). In this same study, males experienced the effects of cocaine faster and with greater intensity than females. In rats, differences in sex hormones and the estrous cycle contribute to differences in sensitivity to cocaine (Becker and Koob 2016; Cao et al. 2018). Thus, sexual dimorphism is a universal feature of the physiological response to cocaine.

Although the *Drosophila* brain is anatomically distinct from the vertebrate brain, fundamental neural processes are evolutionarily conserved. Functions of the dopamine reward pathway in humans are analogous to experience-dependent modulation of behaviors by the mushroom bodies. In our study, ∼69% of genes differentially expressed in response to cocaine have human orthologs (Supplemental Table S3), including *Aldh*, *Dop2R*, *GluRIA*, *GluRIB*, and *Vmat,* previously implicated in cocaine phenotypes. Polymorphisms in the human ortholog for *Aldh*, *ALDH2*, have been associated with drug addiction in Chinese populations (Zhang et al. 2017), and suppression of *ALDH2* suppresses cocaine-seeking behavior (Yao et al. 2010). *Dop2R* encodes a dopamine receptor, and its human ortholog, *DRD2*, is a well-characterized component of the dopamine reward pathway, which mediates development of cocaine dependence (Noble et al. 1993; Persico et al. 1996; Moyer et al. 2011; Stolf et al. 2019). The glutamate receptor genes *GluRIA* and *GluRIB* are associated with glutamatergic neurotransmission, which is altered following exposure to cocaine and has been linked to cocaine sensitization and cocaine-induced behavioral effects (Ghasemzadeh et al. 2011; Liu et al. 2014; Shin et al. 2016). *Vmat* encodes the vesicular monoamine transporter responsible for packaging the neurotransmitters dopamine, serotonin, and octopamine in synaptic vesicles (Greer et al. 2005). In humans, the solute carrier family 18 member A2, SLC18A2 (previously known as VMAT2), has the same function, and protein levels are reduced in cocaine users (Little et al. 2003; Narendran et al. 2014). These functional parallels between the fly model and human studies provide proof-of-principle that results from cocaine exposure obtained from the fly model can be translated to human populations. Thus, the comprehensive documentation of cocaine-mediated modulation of gene expression which we have derived can serve as a contextual framework for future human studies.

## Methods

### *Drosophila* stock

Canton S (B) flies (Norga et al. 2003) were maintained on standard cornmeal/yeast/molasses-agar culture medium at 25°C on a 12:12 h light:dark cycle with 50% humidity in controlled adult density vials to prevent overcrowding. Briefly, five males and five females were placed into a vial and allowed to mate for 2 d before being cleared. Progeny from these vials were collected after eclosion and aged for 3–5 d before experimentation.

### Cocaine exposure

Cocaine.HCl was obtained from the National Institute on Drug Abuse under Drug Enforcement Administration license RA0443159. To expose flies to cocaine, we performed a modified version of the capillary feeder (CAFE) assay (Ja et al. 2007). We collected the first 40 flies that consumed 0.53 µL of cocaine and the first 40 flies that consumed 0.53 µL of sucrose, corresponding to an 8-mm reduction in the height of the solution in the capillary. All experiments were carried out between 8 a.m. and 11 a.m. Flies were allowed to feed for no more than 2 h.

### Behaviors

We measured negative geotaxis and startle response of individual flies within a 10-min time frame immediately following acute exposure to cocaine in the CAFE assay. We quantified grooming and seizures in addition to measuring the behavioral response in each assay. Excessive grooming was defined as more than 10 sec of constant grooming (Supplemental Video S2). Seizure activity was defined as severe muscle tremors that prevented the fly from moving normally (Supplemental Video S3).

### Brain dissection and dissociation

Brains were dissected from each fly immediately after it consumed the designated amount of sucrose or cocaine solution, and we used a dissociation protocol modified from Croset et al. (2018) and Davie et al. (2018). We collected eight samples of 20 brains from males and females exposed to cocaine or sucrose, with two biological replicates per treatment and sex. We proceeded with GEM generation using the Chromium controller (10x Genomics) if we had a live cell count of >500 live cells/µL.

### Library preparation and sequencing

We made libraries after GEM generation in accordance with 10x Genomics v3.1 protocols. We sequenced the final libraries on an S1 flow cell using a NovaSeq (Illumina, Inc.) according to the manufacturer's instructions.

### FASTQ generation, demultiplexing, and alignment

The mkfastq pipeline within Cell Ranger v3.1 (10x Genomics) was used to convert BCL files from the sequence run folder to demultiplexed FASTQ files. Release 6 version of the *Drosophila melanogaster* reference GCA_000001215.4 from the NCBI GenBank database (https://www.ncbi.nlm.nih.gov/genbank/) was indexed using the mkref pipeline and used for alignment using the *count* pipeline within Cell Ranger v3.1 with the expected cell count parameter set to 5000 cells. The sequencing and alignment summary is given in Supplemental Table S2.

### Preprocessing, integration, and cell type clustering

Raw expression counts output for each sample from the Cell Ranger pipeline was imported and analyzed using the Seurat v3 package in R (R core team 2013; Butler et al. 2018). Genes expressed in less than five cells and cells with less than 300 or greater than 2500 RNA features were filtered out. The upper (2500) and lower (300) thresholds for the RNA features per cell were chosen based on the recommendations from the developers of the Seurat v3 pipeline. The recommendation is based on the multitude of observations indicating that cells with less than 300 RNA features tend to have very sparse and unreliable signal and those with greater than 2500 RNA features tend to be miscalled multiplet cells. Normalization and subsequent integration were performed using the sctransform pipeline (Hafemeister and Satija 2019). To identify the cell type clusters within the data set, unsupervised clustering using the FindClusters function and a resolution of 0.8 were used. Cluster marker genes were identified using the FindAllMarker function (min.pct equals; 0.25, logfc.threshold equals; 0.5, only.pos equals; TRUE). The top three genes with positive expression for each cluster were extracted and used for cell type characterization.

### Differential expression

Differential expression was performed for each cluster in two ways: (1) after combining male and female samples together to test for effects of cocaine that are common to both sexes; and (2) testing for effects of cocaine in males and females separately to identify sexually dimorphic responses. The Pearson residuals output from the sctransform pipeline was used as input for differential expression (DE) calculation (Hafemeister and Satija 2019). The MAST algorithm was used as the testing methodology in the FindMarkers function (test.use equals; “MAST”, assay equals; “SCT”, slot equals; “scale.data”) for each cluster to calculate DE. Clusters with a sufficient number of DEGs were subjected to pathway enrichment analysis using the statistical overrepresentation test using PANTHER (Thomas et al. 2003) and Reactome databases (Fabregat et al. 2016). Pathways with BH-FDR adjusted *P*-values < 0.05 were considered statistically enriched.

### Simulation of bulk RNA-seq response

The results from DE calculation from the combined data set were used to determine which genes were consistently up-regulated and down-regulated, respectively, across all clusters as a result of exposure to cocaine. The top 50 ranked differentially up-regulated genes for each cluster and the top 20 ranked differentially down-regulated genes for each cluster were input into the TopKLists R package (Schimek et al. 2015).

### Cluster-specific coexpression networks

The scaled data from the sctransform pipeline for differentially expressed genes from clusters 16 and 22 were extracted for the male samples. These scaled data were used as input for filtering through the Random Matrix Theory (Gibson et al. 2013). The correlations that passed the filtering process were visualized using Cytoscape version 3.7.2. The MCODE algorithm (Bader and Hogue 2003) was utilized to identify highly interconnected modules within the larger cluster network. Genetic interaction networks were constructed by converting the gene IDs to gene names/symbols using the FlyBase Consortium's ‘Query-by-symbols/ID’ tool and calculating interactions between gene products using the StringApp plugin within Cytoscape (Doncheva et al. 2019). To identify specific pathways that are enriched in genes within each of the circular groups, we performed statistical overrepresentation tests on the gene IDs from each group using PANTHER (Thomas et al. 2003) and Reactome (Fabregat et al. 2018) databases. Pathways with BH-FDR adjusted *P*-values < 0.05 were considered statistically enriched.

## Data access

All single-cell RNA sequences data generated in this study have been submitted to the NCBI Gene Expression Omnibus (GEO; https://www.ncbi.nlm.nih.gov/geo/) under accession number GSE152495. R code that was used to perform Seurat-based analyses with TopKLists is included in Supplemental Code and is available at GitHub (https://github.com/vshanka23/The-Drosophila-Brain-on-Cocaine-at-Single-Cell-Resolution/blob/master/Rcode_for_analyses.R).

## Supplementary Material

Supplemental Material
