# Supplementary material for: The Drosophila brain on cocaine at single-cell resolution
Source: Genome Res. 2021 Oct;31(10):1927–37. doi: 10.1101/gr.268037.120 (PMC8494231; doi:10.1101/gr.268037.120)
Supplement: Supplemental Material [file supp_31_10_1927__DC1.html]

The Drosophila brain on cocaine at single-cell resolution — Supplemental Material 

# The *Drosophila* brain on cocaine at single-cell resolution

## Supplemental Material

- Supplemental\_Table\_S1\_xlsx.xlsx
- Supplemental\_Table\_S3\_xlsx.xlsx
- Supplemental\_Table\_S5\_xlsx.xlsx
- Supplemental\_Table\_S6\_xlsx.xlsx
- Supplemental\_Table\_S7\_xlsx.xlsx
- Supplemental\_Table\_S8\_xlsx.xlsx
- Supplemental\_Table\_S10\_xlsx.xlsx
- Supplemental\_Movie\_S2\_mp4.mp4
- Supplemental\_Movie\_S3\_mp4.mp4
- Supplemental\_code.docx
- Supplemental\_Material.pdf
- Supplemental\_Table\_S9\_xlsx.xlsx
- Supelemental\_Movie\_S1\_mp4.mp4
