# Supplementary material for: The Drosophila brain on cocaine at single-cell resolution
Source: Genome Res. 2021 Oct;31(10):1927–37. doi: 10.1101/gr.268037.120 (PMC8494231; doi:10.1101/gr.268037.120)
Supplement: Supplemental Material [file supp_gr.268037.120_Supplemental_code.docx]

**Supplemental Code**

library(cowplot)

library(ggplot2)

library(dplyr)

library(Seurat)

library(rlang)

library(TopKLists)

library(readxl)

##scTransform requires considerable RAM so add this statement before proceeding to intergration.

options(future.globals.maxSize= 53687091200)

##Read data in

Female_sucrose_R1_Data <- Read10X(data.dir="/CHG_backup2/Sequencing_data/Single_cell_sequencing/Brain_cocaine/Run1/2_counts_31/S1/outs/filtered_feature_bc_matrix/")

Female_sucrose_R2_Data <- Read10X(data.dir="/CHG_backup2/Sequencing_data/Single_cell_sequencing/Brain_cocaine/Run1/2_counts_31/S2/outs/filtered_feature_bc_matrix/")

Male_sucrose_R1_Data <- Read10X(data.dir="/CHG_backup2/Sequencing_data/Single_cell_sequencing/Brain_cocaine/Run1/2_counts_31/S3/outs/filtered_feature_bc_matrix/")

Male_sucrose_R2_Data <- Read10X(data.dir="/CHG_backup2/Sequencing_data/Single_cell_sequencing/Brain_cocaine/Run1/2_counts_31/S4/outs/filtered_feature_bc_matrix/")

Female_cocaine_R1_Data <- Read10X(data.dir="/CHG_backup2/Sequencing_data/Single_cell_sequencing/Brain_cocaine/Run1/2_counts_31/S5/outs/filtered_feature_bc_matrix/")

Female_cocaine_R2_Data <- Read10X(data.dir="/CHG_backup2/Sequencing_data/Single_cell_sequencing/Brain_cocaine/Run1/2_counts_31/S6/outs/filtered_feature_bc_matrix/")

Male_cocaine_R1_Data <- Read10X(data.dir="/CHG_backup2/Sequencing_data/Single_cell_sequencing/Brain_cocaine/Run1/2_counts_31/S7/outs/filtered_feature_bc_matrix/")

Male_cocaine_R2_Data <- Read10X(data.dir="/CHG_backup2/Sequencing_data/Single_cell_sequencing/Brain_cocaine/Run1/2_counts_31/S8v2/outs/filtered_feature_bc_matrix/")

##Create Seurat objects

Female_sucrose_R1 <- CreateSeuratObject(counts=Female_sucrose_R1_Data,project="Female_Sucrose",min.cells=5)

Female_sucrose_R2 <- CreateSeuratObject(counts=Female_sucrose_R2_Data,project="Female_Sucrose",min.cells=5)

Male_sucrose_R1 <- CreateSeuratObject(counts=Male_sucrose_R1_Data,project="Male_Sucrose",min.cells = 5)

Male_sucrose_R2 <- CreateSeuratObject(counts=Male_sucrose_R2_Data,project="Male_Sucrose",min.cells = 5)

Female_cocaine_R2 <- CreateSeuratObject(counts=Female_cocaine_R2_Data,project="Female_Cocaine",min.cells=5)

Female_cocaine_R1 <- CreateSeuratObject(counts=Female_cocaine_R1_Data,project="Female_Cocaine",min.cells=5)

Male_cocaine_R1 <- CreateSeuratObject(counts=Male_cocaine_R1_Data,project="Male_Cocaine",min.cells = 5)

Male_cocaine_R2 <- CreateSeuratObject(counts=Male_cocaine_R2_Data,project="Male_Cocaine",min.cells = 5)

##Set identities for condition

Male_cocaine_R1$stim <- "cocaine"

Male_cocaine_R2$stim <- "cocaine"

Male_sucrose_R1$stim <- "sucrose"

Male_sucrose_R2$stim <- "sucrose"

Female_cocaine_R1$stim <- "cocaine"

Female_cocaine_R2$stim <- "cocaine"

Female_sucrose_R1$stim <- "sucrose"

Female_sucrose_R2$stim <- "sucrose"

##Set identities for gender and condition

Male_cocaine_R1$gender_stim <- "male_cocaine"

Male_cocaine_R2$gender_stim <- "male_cocaine"

Male_sucrose_R1$gender_stim <- "male_sucrose"

Male_sucrose_R2$gender_stim <- "male_sucrose"

Female_cocaine_R1$gender_stim <- "female_cocaine"

Female_cocaine_R2$gender_stim <- "female_cocaine"

Female_sucrose_R1$gender_stim <- "female_sucrose"

Female_sucrose_R2$gender_stim <- "female_sucrose"

##Set identities for sample

Male_cocaine_R1$sample_id <- "male_cocaine_R1"

Male_cocaine_R2$sample_id <- "male_cocaine_R2"

Male_sucrose_R1$sample_id <- "male_sucrose_R1"

Male_sucrose_R2$sample_id <- "male_sucrose_R2"

Female_cocaine_R1$sample_id <- "female_cocaine_R1"

Female_cocaine_R2$sample_id <- "female_cocaine_R2"

Female_sucrose_R1$sample_id <- "female_sucrose_R1"

Female_sucrose_R2$sample_id <- "female_sucrose_R2"

##Remove spurious features (low and high)

Male_cocaine_R2 <- subset(Male_cocaine_R2,subset=nFeature_RNA > 300 & nFeature_RNA < 2500)

Male_cocaine_R1 <- subset(Male_cocaine_R1,subset=nFeature_RNA > 300 & nFeature_RNA < 2500)

Male_sucrose_R2 <- subset(Male_sucrose_R2,subset=nFeature_RNA > 300 & nFeature_RNA <2500)

Male_sucrose_R1 <- subset(Male_sucrose_R1,subset=nFeature_RNA > 300 & nFeature_RNA <2500)

Female_cocaine_R2 <- subset(Female_cocaine_R2,subset=nFeature_RNA > 300 & nFeature_RNA < 2500)

Female_cocaine_R1 <- subset(Female_cocaine_R1,subset=nFeature_RNA > 300 & nFeature_RNA < 2500)

Female_sucrose_R2 <- subset(Female_sucrose_R2,subset=nFeature_RNA > 300 & nFeature_RNA < 2500)

Female_sucrose_R1 <- subset(Female_sucrose_R1,subset=nFeature_RNA > 300 & nFeature_RNA < 2500)

##Normalize

Male_cocaine_R1 <- SCTransform(Male_cocaine_R1,verbose = FALSE,return.only.var.genes = FALSE)

Male_cocaine_R2 <- SCTransform(Male_cocaine_R2,verbose = FALSE,return.only.var.genes = FALSE)

Male_sucrose_R1 <- SCTransform(Male_sucrose_R1,verbose = FALSE,return.only.var.genes = FALSE)

Male_sucrose_R2 <- SCTransform(Male_sucrose_R2,verbose = FALSE,return.only.var.genes = FALSE)

Female_cocaine_R1 <- SCTransform(Female_cocaine_R1,verbose = FALSE,return.only.var.genes = FALSE)

Female_cocaine_R2 <- SCTransform(Female_cocaine_R2,verbose = FALSE,return.only.var.genes = FALSE)

Female_sucrose_R1 <- SCTransform(Female_sucrose_R1,verbose = FALSE,return.only.var.genes = FALSE)

Female_sucrose_R2 <- SCTransform(Female_sucrose_R2,verbose = FALSE,return.only.var.genes = FALSE)

##Feature selection

Brain.features <- SelectIntegrationFeatures(object.list=c(Male_cocaine_R1,Male_cocaine_R2,Male_sucrose_R1,Male_sucrose_R2,Female_cocaine_R1,Female_cocaine_R2,Female_sucrose_R1,Female_sucrose_R2),nfeatures=1500)

Brain.list <- PrepSCTIntegration(object.list = c(Male_cocaine_R1,Male_cocaine_R2,Male_sucrose_R1,Male_sucrose_R2,Female_cocaine_R1,Female_cocaine_R2,Female_sucrose_R1,Female_sucrose_R2),anchor.features = Brain.features,verbose = FALSE)

##Identify anchors

Brain.anchors <- FindIntegrationAnchors(object.list = Brain.list, normalization.method = "SCT", anchor.features = Brain.features, verbose = FALSE)

Brain.integrated <- IntegrateData(anchorset = Brain.anchors, normalization.method = "SCT", verbose = FALSE)

Brain.integrated <- RunPCA(object = Brain.integrated, verbose = FALSE)

##Break - Use elbowplot to determine how many dimensions to use for capturing as much variability as possible without using all of the axes. Look for a point of desaturation (elbow).

ElbowPlot(Brain.integrated)

##Break

##Use info from elbow plot to set number of dimensions for UMAP

Brain.integrated <- RunUMAP(Brain.integrated, reduction = "pca", dims = 1:10)

Brain.integrated <- FindNeighbors(Brain.integrated,reduction = "pca", dims = 1:10)

##Cluster cells, but first find the correct resolution by trying to apply a range of values

brain_clusters <- FindClusters(Brain.integrated,resolution = 0.4)

brain_clusters <- FindClusters(Brain.integrated,resolution = 0.5)

brain_clusters <- FindClusters(Brain.integrated,resolution = 0.6)

brain_clusters <- FindClusters(Brain.integrated,resolution = 0.7)

brain_clusters <- FindClusters(Brain.integrated,resolution = 0.8)

brain_clusters <- FindClusters(Brain.integrated,resolution = 0.9)

brain_clusters <- FindClusters(Brain.integrated,resolution = 1.0)

brain_clusters <- FindClusters(Brain.integrated,resolution = 1.1)

brain_clusters <- FindClusters(Brain.integrated,resolution = 1.2)

brain_clusters <- FindClusters(Brain.integrated,resolution = 1.3)

brain_clusters <- FindClusters(Brain.integrated,resolution = 1.4)

brain_clusters <- FindClusters(Brain.integrated,resolution = 1.5)

brain_clusters <- FindClusters(Brain.integrated,resolution = 1.6)

brain_clusters <- FindClusters(Brain.integrated,resolution = 1.7)

brain_clusters <- FindClusters(Brain.integrated,resolution = 1.8)

brain_clusters <- FindClusters(Brain.integrated,resolution = 1.9)

brain_clusters <- FindClusters(Brain.integrated,resolution = 2.0)

##Once a suitable resolution has been found, rerun the FindCluster with that value.

Brain.integrated <- FindClusters(Brain.integrated, resolution = 0.8)

Brain.integrated$Celltype <- Idents(Brain.integrated)

plots <- DimPlot(Brain.integrated,reduction="umap",group.by=c("stim","gender_stim","Celltype","sample_id"),combine=FALSE)

plots <- lapply(X = plots, FUN = function(x) + theme(legend.position = "top") + guides(color = guide_legend(nrow = 2), byrow= TRUE, override.aes = list(size=3))))

CombinePlots(plots)

##Create new identities for DE analysis downstream

Brain.integrated$celltype.stim <- paste(Idents(Brain.integrated),Brain.integrated$stim,sep="_")

Brain.integrated$celltype.gender_stim <- paste(Idents(Brain.integrated),Brain.integrated$gender_stim,sep="_")

##Switch to RNA assay to find cluster markers

DefaultAssay(Brain.integrated) <- "RNA"

Brain.integrated <- NormalizeData(Brain.integrated, verbose = FALSE)

Brain.integrated_markers_all <- FindAllMarkers(Brain.integrated, min.pct=0.25, logfc.threshold = 0.5, only.pos = TRUE)

Brain.integrated_cluster_markers <- Brain.integrated_markers_all %>% group_by(cluster) %>% top_n(n=3, wt = avg_logFC) %>% print(n=3*37)

write.csv(Brain.integrated_cluster_markers, "Brain_integrated_cluster_markers_top3.csv")

##Change labels to marker genes

new.cluster.ids <- c("VGlut/CG2269/CG32017","Gad1/CG14989/CG32017","spab/jeb/CG31221","VGlut/CG34355/CG9650","SoxN/CG9650/klg","pros/dati/spab","toy/bi","pros/br/beat-IIIC","sosie/acj6/nAChRalpha6","Gad1/Lim3/CG14989","mbl/Imp/CG31345","jdp/Pka-R2/Rgk1","ct/Gad1/CG14989","Obp44a/CG8369/CG15201","pdm3/vvl/CG18598","Pka-C3/sosie/CG2016","CNMaR/SoxN/CG42750","Eaat1/CG2016/CG15522","DIP-theta/aop/CG42458","Gs2/CG1552/CG8369","Pka-R2/crb/Rgk1","VGlut/mbl/Proc","Cys/SPARC/CG3168","Nos/Octbeta1R/CG14989","Vmat/Hsp23/CG4577","bi/shakB/Octbeta1R","shakB/CG3134/CG18598","Dh31/Nep1/CG14687","Frq1/CG43795/CG14274","DAT/Vmat/ple","Arr2/Arr1/ninaE","CG2016/CG6044/CG42540","kn/sNPF/lncR:CR45223","VGlut/Ca-alpha1T/CG8034","Tk/Nplp1/nAChRalpha6","sNPF/CG14989/lncR:CR45223")

names(new.cluster.ids)<-levels(Brain.integrated)

Brain.integrated<-RenameIdents(Brain.integrated,new.cluster.ids)

DefaultAssay(Brain.integrated) <-"integrated"

DimPlot(Brain.integrated,label = TRUE) + NoLegend()

##Perform DE

##Store previous biological cluster identities

Brain.integrated$cluster_ids <- Idents(Brain.integrated)

##replace with gender + condition based identities

Idents(Brain.integrated) <- "celltype.gender_stim"

##Test female first

Brain_cocaine_DE_female_stim_C0 <- FindMarkers(Brain.integrated,ident.1 = "0_female_cocaine",ident.2 = "0_female_sucrose",test.use = "MAST", assay = "SCT", slot = "scale.data")

Brain_cocaine_DE_female_stim_C1 <- FindMarkers(Brain.integrated,ident.1 = "1_female_cocaine",ident.2 = "1_female_sucrose",test.use = "MAST", assay = "SCT", slot = "scale.data")

Brain_cocaine_DE_female_stim_C2 <- FindMarkers(Brain.integrated,ident.1 = "2_female_cocaine",ident.2 = "2_female_sucrose",test.use = "MAST", assay = "SCT", slot = "scale.data")

Brain_cocaine_DE_female_stim_C3 <- FindMarkers(Brain.integrated,ident.1 = "3_female_cocaine",ident.2 = "3_female_sucrose",test.use = "MAST", assay = "SCT", slot = "scale.data")

Brain_cocaine_DE_female_stim_C4 <- FindMarkers(Brain.integrated,ident.1 = "4_female_cocaine",ident.2 = "4_female_sucrose",test.use = "MAST", assay = "SCT", slot = "scale.data")

Brain_cocaine_DE_female_stim_C5 <- FindMarkers(Brain.integrated,ident.1 = "5_female_cocaine",ident.2 = "5_female_sucrose",test.use = "MAST", assay = "SCT", slot = "scale.data")

Brain_cocaine_DE_female_stim_C6 <- FindMarkers(Brain.integrated,ident.1 = "6_female_cocaine",ident.2 = "6_female_sucrose",test.use = "MAST", assay = "SCT", slot = "scale.data")

Brain_cocaine_DE_female_stim_C7 <- FindMarkers(Brain.integrated,ident.1 = "7_female_cocaine",ident.2 = "7_female_sucrose",test.use = "MAST", assay = "SCT", slot = "scale.data")

Brain_cocaine_DE_female_stim_C8 <- FindMarkers(Brain.integrated,ident.1 = "8_female_cocaine",ident.2 = "8_female_sucrose",test.use = "MAST", assay = "SCT", slot = "scale.data")

Brain_cocaine_DE_female_stim_C9 <- FindMarkers(Brain.integrated,ident.1 = "9_female_cocaine",ident.2 = "9_female_sucrose",test.use = "MAST", assay = "SCT", slot = "scale.data")

Brain_cocaine_DE_female_stim_C10 <- FindMarkers(Brain.integrated,ident.1 = "10_female_cocaine",ident.2 = "10_female_sucrose",test.use = "MAST", assay = "SCT", slot = "scale.data")

Brain_cocaine_DE_female_stim_C11 <- FindMarkers(Brain.integrated,ident.1 = "11_female_cocaine",ident.2 = "11_female_sucrose",test.use = "MAST", assay = "SCT", slot = "scale.data")

Brain_cocaine_DE_female_stim_C12 <- FindMarkers(Brain.integrated,ident.1 = "12_female_cocaine",ident.2 = "12_female_sucrose",test.use = "MAST", assay = "SCT", slot = "scale.data")

Brain_cocaine_DE_female_stim_C13 <- FindMarkers(Brain.integrated,ident.1 = "13_female_cocaine",ident.2 = "13_female_sucrose",test.use = "MAST", assay = "SCT", slot = "scale.data")

Brain_cocaine_DE_female_stim_C14 <- FindMarkers(Brain.integrated,ident.1 = "14_female_cocaine",ident.2 = "14_female_sucrose",test.use = "MAST", assay = "SCT", slot = "scale.data")

Brain_cocaine_DE_female_stim_C15 <- FindMarkers(Brain.integrated,ident.1 = "15_female_cocaine",ident.2 = "15_female_sucrose",test.use = "MAST", assay = "SCT", slot = "scale.data")

Brain_cocaine_DE_female_stim_C16 <- FindMarkers(Brain.integrated,ident.1 = "16_female_cocaine",ident.2 = "16_female_sucrose",test.use = "MAST", assay = "SCT", slot = "scale.data")

Brain_cocaine_DE_female_stim_C17 <- FindMarkers(Brain.integrated,ident.1 = "17_female_cocaine",ident.2 = "17_female_sucrose",test.use = "MAST", assay = "SCT", slot = "scale.data")

Brain_cocaine_DE_female_stim_C18 <- FindMarkers(Brain.integrated,ident.1 = "18_female_cocaine",ident.2 = "18_female_sucrose",test.use = "MAST", assay = "SCT", slot = "scale.data")

Brain_cocaine_DE_female_stim_C19 <- FindMarkers(Brain.integrated,ident.1 = "19_female_cocaine",ident.2 = "19_female_sucrose",test.use = "MAST", assay = "SCT", slot = "scale.data")

Brain_cocaine_DE_female_stim_C20 <- FindMarkers(Brain.integrated,ident.1 = "20_female_cocaine",ident.2 = "20_female_sucrose",test.use = "MAST", assay = "SCT", slot = "scale.data")

Brain_cocaine_DE_female_stim_C21 <- FindMarkers(Brain.integrated,ident.1 = "21_female_cocaine",ident.2 = "21_female_sucrose",test.use = "MAST", assay = "SCT", slot = "scale.data")

Brain_cocaine_DE_female_stim_C22 <- FindMarkers(Brain.integrated,ident.1 = "22_female_cocaine",ident.2 = "22_female_sucrose",test.use = "MAST", assay = "SCT", slot = "scale.data")

Brain_cocaine_DE_female_stim_C23 <- FindMarkers(Brain.integrated,ident.1 = "23_female_cocaine",ident.2 = "23_female_sucrose",test.use = "MAST", assay = "SCT", slot = "scale.data")

Brain_cocaine_DE_female_stim_C24 <- FindMarkers(Brain.integrated,ident.1 = "24_female_cocaine",ident.2 = "24_female_sucrose",test.use = "MAST", assay = "SCT", slot = "scale.data")

Brain_cocaine_DE_female_stim_C25 <- FindMarkers(Brain.integrated,ident.1 = "25_female_cocaine",ident.2 = "25_female_sucrose",test.use = "MAST", assay = "SCT", slot = "scale.data")

Brain_cocaine_DE_female_stim_C26 <- FindMarkers(Brain.integrated,ident.1 = "26_female_cocaine",ident.2 = "26_female_sucrose",test.use = "MAST", assay = "SCT", slot = "scale.data")

Brain_cocaine_DE_female_stim_C27 <- FindMarkers(Brain.integrated,ident.1 = "27_female_cocaine",ident.2 = "27_female_sucrose",test.use = "MAST", assay = "SCT", slot = "scale.data")

Brain_cocaine_DE_female_stim_C28 <- FindMarkers(Brain.integrated,ident.1 = "28_female_cocaine",ident.2 = "28_female_sucrose",test.use = "MAST", assay = "SCT", slot = "scale.data")

Brain_cocaine_DE_female_stim_C29 <- FindMarkers(Brain.integrated,ident.1 = "29_female_cocaine",ident.2 = "29_female_sucrose",test.use = "MAST", assay = "SCT", slot = "scale.data")

Brain_cocaine_DE_female_stim_C30 <- FindMarkers(Brain.integrated,ident.1 = "30_female_cocaine",ident.2 = "30_female_sucrose",test.use = "MAST", assay = "SCT", slot = "scale.data")

Brain_cocaine_DE_female_stim_C31 <- FindMarkers(Brain.integrated,ident.1 = "31_female_cocaine",ident.2 = "31_female_sucrose",test.use = "MAST", assay = "SCT", slot = "scale.data")

Brain_cocaine_DE_female_stim_C32 <- FindMarkers(Brain.integrated,ident.1 = "32_female_cocaine",ident.2 = "32_female_sucrose",test.use = "MAST", assay = "SCT", slot = "scale.data")

Brain_cocaine_DE_female_stim_C33 <- FindMarkers(Brain.integrated,ident.1 = "33_female_cocaine",ident.2 = "33_female_sucrose",test.use = "MAST", assay = "SCT", slot = "scale.data")

Brain_cocaine_DE_female_stim_C34 <- FindMarkers(Brain.integrated,ident.1 = "34_female_cocaine",ident.2 = "34_female_sucrose",test.use = "MAST", assay = "SCT", slot = "scale.data")

Brain_cocaine_DE_female_stim_C35 <- FindMarkers(Brain.integrated,ident.1 = "35_female_cocaine",ident.2 = "35_female_sucrose",test.use = "MAST", assay = "SCT", slot = "scale.data")

##test male second

Brain_cocaine_DE_male_stim_C0 <- FindMarkers(Brain.integrated,ident.1 = "0_male_cocaine",ident.2 = "0_male_sucrose",test.use = "MAST", assay = "SCT", slot = "scale.data")

Brain_cocaine_DE_male_stim_C1 <- FindMarkers(Brain.integrated,ident.1 = "1_male_cocaine",ident.2 = "1_male_sucrose",test.use = "MAST", assay = "SCT", slot = "scale.data")

Brain_cocaine_DE_male_stim_C2 <- FindMarkers(Brain.integrated,ident.1 = "2_male_cocaine",ident.2 = "2_male_sucrose",test.use = "MAST", assay = "SCT", slot = "scale.data")

Brain_cocaine_DE_male_stim_C3 <- FindMarkers(Brain.integrated,ident.1 = "3_male_cocaine",ident.2 = "3_male_sucrose",test.use = "MAST", assay = "SCT", slot = "scale.data")

Brain_cocaine_DE_male_stim_C4 <- FindMarkers(Brain.integrated,ident.1 = "4_male_cocaine",ident.2 = "4_male_sucrose",test.use = "MAST", assay = "SCT", slot = "scale.data")

Brain_cocaine_DE_male_stim_C5 <- FindMarkers(Brain.integrated,ident.1 = "5_male_cocaine",ident.2 = "5_male_sucrose",test.use = "MAST", assay = "SCT", slot = "scale.data")

Brain_cocaine_DE_male_stim_C6 <- FindMarkers(Brain.integrated,ident.1 = "6_male_cocaine",ident.2 = "6_male_sucrose",test.use = "MAST", assay = "SCT", slot = "scale.data")

Brain_cocaine_DE_male_stim_C7 <- FindMarkers(Brain.integrated,ident.1 = "7_male_cocaine",ident.2 = "7_male_sucrose",test.use = "MAST", assay = "SCT", slot = "scale.data")

Brain_cocaine_DE_male_stim_C8 <- FindMarkers(Brain.integrated,ident.1 = "8_male_cocaine",ident.2 = "8_male_sucrose",test.use = "MAST", assay = "SCT", slot = "scale.data")

Brain_cocaine_DE_male_stim_C9 <- FindMarkers(Brain.integrated,ident.1 = "9_male_cocaine",ident.2 = "9_male_sucrose",test.use = "MAST", assay = "SCT", slot = "scale.data")

Brain_cocaine_DE_male_stim_C10 <- FindMarkers(Brain.integrated,ident.1 = "10_male_cocaine",ident.2 = "10_male_sucrose",test.use = "MAST", assay = "SCT", slot = "scale.data")

Brain_cocaine_DE_male_stim_C11 <- FindMarkers(Brain.integrated,ident.1 = "11_male_cocaine",ident.2 = "11_male_sucrose",test.use = "MAST", assay = "SCT", slot = "scale.data")

Brain_cocaine_DE_male_stim_C12 <- FindMarkers(Brain.integrated,ident.1 = "12_male_cocaine",ident.2 = "12_male_sucrose",test.use = "MAST", assay = "SCT", slot = "scale.data")

Brain_cocaine_DE_male_stim_C13 <- FindMarkers(Brain.integrated,ident.1 = "13_male_cocaine",ident.2 = "13_male_sucrose",test.use = "MAST", assay = "SCT", slot = "scale.data")

Brain_cocaine_DE_male_stim_C14 <- FindMarkers(Brain.integrated,ident.1 = "14_male_cocaine",ident.2 = "14_male_sucrose",test.use = "MAST", assay = "SCT", slot = "scale.data")

Brain_cocaine_DE_male_stim_C15 <- FindMarkers(Brain.integrated,ident.1 = "15_male_cocaine",ident.2 = "15_male_sucrose",test.use = "MAST", assay = "SCT", slot = "scale.data")

Brain_cocaine_DE_male_stim_C16 <- FindMarkers(Brain.integrated,ident.1 = "16_male_cocaine",ident.2 = "16_male_sucrose",test.use = "MAST", assay = "SCT", slot = "scale.data")

Brain_cocaine_DE_male_stim_C17 <- FindMarkers(Brain.integrated,ident.1 = "17_male_cocaine",ident.2 = "17_male_sucrose",test.use = "MAST", assay = "SCT", slot = "scale.data")

Brain_cocaine_DE_male_stim_C18 <- FindMarkers(Brain.integrated,ident.1 = "18_male_cocaine",ident.2 = "18_male_sucrose",test.use = "MAST", assay = "SCT", slot = "scale.data")

Brain_cocaine_DE_male_stim_C19 <- FindMarkers(Brain.integrated,ident.1 = "19_male_cocaine",ident.2 = "19_male_sucrose",test.use = "MAST", assay = "SCT", slot = "scale.data")

Brain_cocaine_DE_male_stim_C20 <- FindMarkers(Brain.integrated,ident.1 = "20_male_cocaine",ident.2 = "20_male_sucrose",test.use = "MAST", assay = "SCT", slot = "scale.data")

Brain_cocaine_DE_male_stim_C21 <- FindMarkers(Brain.integrated,ident.1 = "21_male_cocaine",ident.2 = "21_male_sucrose",test.use = "MAST", assay = "SCT", slot = "scale.data")

Brain_cocaine_DE_male_stim_C22 <- FindMarkers(Brain.integrated,ident.1 = "22_male_cocaine",ident.2 = "22_male_sucrose",test.use = "MAST", assay = "SCT", slot = "scale.data")

Brain_cocaine_DE_male_stim_C23 <- FindMarkers(Brain.integrated,ident.1 = "23_male_cocaine",ident.2 = "23_male_sucrose",test.use = "MAST", assay = "SCT", slot = "scale.data")

Brain_cocaine_DE_male_stim_C24 <- FindMarkers(Brain.integrated,ident.1 = "24_male_cocaine",ident.2 = "24_male_sucrose",test.use = "MAST", assay = "SCT", slot = "scale.data")

Brain_cocaine_DE_male_stim_C25 <- FindMarkers(Brain.integrated,ident.1 = "25_male_cocaine",ident.2 = "25_male_sucrose",test.use = "MAST", assay = "SCT", slot = "scale.data")

Brain_cocaine_DE_male_stim_C26 <- FindMarkers(Brain.integrated,ident.1 = "26_male_cocaine",ident.2 = "26_male_sucrose",test.use = "MAST", assay = "SCT", slot = "scale.data")

Brain_cocaine_DE_male_stim_C27 <- FindMarkers(Brain.integrated,ident.1 = "27_male_cocaine",ident.2 = "27_male_sucrose",test.use = "MAST", assay = "SCT", slot = "scale.data")

Brain_cocaine_DE_male_stim_C28 <- FindMarkers(Brain.integrated,ident.1 = "28_male_cocaine",ident.2 = "28_male_sucrose",test.use = "MAST", assay = "SCT", slot = "scale.data")

Brain_cocaine_DE_male_stim_C29 <- FindMarkers(Brain.integrated,ident.1 = "29_male_cocaine",ident.2 = "29_male_sucrose",test.use = "MAST", assay = "SCT", slot = "scale.data")

Brain_cocaine_DE_male_stim_C30 <- FindMarkers(Brain.integrated,ident.1 = "30_male_cocaine",ident.2 = "30_male_sucrose",test.use = "MAST", assay = "SCT", slot = "scale.data")

Brain_cocaine_DE_male_stim_C31 <- FindMarkers(Brain.integrated,ident.1 = "31_male_cocaine",ident.2 = "31_male_sucrose",test.use = "MAST", assay = "SCT", slot = "scale.data")

Brain_cocaine_DE_male_stim_C32 <- FindMarkers(Brain.integrated,ident.1 = "32_male_cocaine",ident.2 = "32_male_sucrose",test.use = "MAST", assay = "SCT", slot = "scale.data")

Brain_cocaine_DE_male_stim_C33 <- FindMarkers(Brain.integrated,ident.1 = "33_male_cocaine",ident.2 = "33_male_sucrose",test.use = "MAST", assay = "SCT", slot = "scale.data")

Brain_cocaine_DE_male_stim_C34 <- FindMarkers(Brain.integrated,ident.1 = "34_male_cocaine",ident.2 = "34_male_sucrose",test.use = "MAST", assay = "SCT", slot = "scale.data")

Brain_cocaine_DE_male_stim_C35 <- FindMarkers(Brain.integrated,ident.1 = "35_male_cocaine",ident.2 = "35_male_sucrose",test.use = "MAST", assay = "SCT", slot = "scale.data")

##Next try globally (meaning across both male and female)

Idents(Brain.integrated) <- "celltype.stim"

Brain_cocaine_DE_stim_C0 <- FindMarkers(Brain.integrated,ident.1 = "0_cocaine",ident.2 = "0_sucrose",test.use = "MAST", assay = "SCT", slot = "scale.data")

Brain_cocaine_DE_stim_C1 <- FindMarkers(Brain.integrated,ident.1 = "1_cocaine",ident.2 = "1_sucrose",test.use = "MAST", assay = "SCT", slot = "scale.data")

Brain_cocaine_DE_stim_C2 <- FindMarkers(Brain.integrated,ident.1 = "2_cocaine",ident.2 = "2_sucrose",test.use = "MAST", assay = "SCT", slot = "scale.data")

Brain_cocaine_DE_stim_C3 <- FindMarkers(Brain.integrated,ident.1 = "3_cocaine",ident.2 = "3_sucrose",test.use = "MAST", assay = "SCT", slot = "scale.data")

Brain_cocaine_DE_stim_C4 <- FindMarkers(Brain.integrated,ident.1 = "4_cocaine",ident.2 = "4_sucrose",test.use = "MAST", assay = "SCT", slot = "scale.data")

Brain_cocaine_DE_stim_C5 <- FindMarkers(Brain.integrated,ident.1 = "5_cocaine",ident.2 = "5_sucrose",test.use = "MAST", assay = "SCT", slot = "scale.data")

Brain_cocaine_DE_stim_C6 <- FindMarkers(Brain.integrated,ident.1 = "6_cocaine",ident.2 = "6_sucrose",test.use = "MAST", assay = "SCT", slot = "scale.data")

Brain_cocaine_DE_stim_C7 <- FindMarkers(Brain.integrated,ident.1 = "7_cocaine",ident.2 = "7_sucrose",test.use = "MAST", assay = "SCT", slot = "scale.data")

Brain_cocaine_DE_stim_C8 <- FindMarkers(Brain.integrated,ident.1 = "8_cocaine",ident.2 = "8_sucrose",test.use = "MAST", assay = "SCT", slot = "scale.data")

Brain_cocaine_DE_stim_C9 <- FindMarkers(Brain.integrated,ident.1 = "9_cocaine",ident.2 = "9_sucrose",test.use = "MAST", assay = "SCT", slot = "scale.data")

Brain_cocaine_DE_stim_C10 <- FindMarkers(Brain.integrated,ident.1 = "10_cocaine",ident.2 = "10_sucrose",test.use = "MAST", assay = "SCT", slot = "scale.data")

Brain_cocaine_DE_stim_C11 <- FindMarkers(Brain.integrated,ident.1 = "11_cocaine",ident.2 = "11_sucrose",test.use = "MAST", assay = "SCT", slot = "scale.data")

Brain_cocaine_DE_stim_C12 <- FindMarkers(Brain.integrated,ident.1 = "12_cocaine",ident.2 = "12_sucrose",test.use = "MAST", assay = "SCT", slot = "scale.data")

Brain_cocaine_DE_stim_C13 <- FindMarkers(Brain.integrated,ident.1 = "13_cocaine",ident.2 = "13_sucrose",test.use = "MAST", assay = "SCT", slot = "scale.data")

Brain_cocaine_DE_stim_C14 <- FindMarkers(Brain.integrated,ident.1 = "14_cocaine",ident.2 = "14_sucrose",test.use = "MAST", assay = "SCT", slot = "scale.data")

Brain_cocaine_DE_stim_C15 <- FindMarkers(Brain.integrated,ident.1 = "15_cocaine",ident.2 = "15_sucrose",test.use = "MAST", assay = "SCT", slot = "scale.data")

Brain_cocaine_DE_stim_C16 <- FindMarkers(Brain.integrated,ident.1 = "16_cocaine",ident.2 = "16_sucrose",test.use = "MAST", assay = "SCT", slot = "scale.data")

Brain_cocaine_DE_stim_C17 <- FindMarkers(Brain.integrated,ident.1 = "17_cocaine",ident.2 = "17_sucrose",test.use = "MAST", assay = "SCT", slot = "scale.data")

Brain_cocaine_DE_stim_C18 <- FindMarkers(Brain.integrated,ident.1 = "18_cocaine",ident.2 = "18_sucrose",test.use = "MAST", assay = "SCT", slot = "scale.data")

Brain_cocaine_DE_stim_C19 <- FindMarkers(Brain.integrated,ident.1 = "19_cocaine",ident.2 = "19_sucrose",test.use = "MAST", assay = "SCT", slot = "scale.data")

Brain_cocaine_DE_stim_C20 <- FindMarkers(Brain.integrated,ident.1 = "20_cocaine",ident.2 = "20_sucrose",test.use = "MAST", assay = "SCT", slot = "scale.data")

Brain_cocaine_DE_stim_C21 <- FindMarkers(Brain.integrated,ident.1 = "21_cocaine",ident.2 = "21_sucrose",test.use = "MAST", assay = "SCT", slot = "scale.data")

Brain_cocaine_DE_stim_C22 <- FindMarkers(Brain.integrated,ident.1 = "22_cocaine",ident.2 = "22_sucrose",test.use = "MAST", assay = "SCT", slot = "scale.data")

Brain_cocaine_DE_stim_C23 <- FindMarkers(Brain.integrated,ident.1 = "23_cocaine",ident.2 = "23_sucrose",test.use = "MAST", assay = "SCT", slot = "scale.data")

Brain_cocaine_DE_stim_C24 <- FindMarkers(Brain.integrated,ident.1 = "24_cocaine",ident.2 = "24_sucrose",test.use = "MAST", assay = "SCT", slot = "scale.data")

Brain_cocaine_DE_stim_C25 <- FindMarkers(Brain.integrated,ident.1 = "25_cocaine",ident.2 = "25_sucrose",test.use = "MAST", assay = "SCT", slot = "scale.data")

Brain_cocaine_DE_stim_C26 <- FindMarkers(Brain.integrated,ident.1 = "26_cocaine",ident.2 = "26_sucrose",test.use = "MAST", assay = "SCT", slot = "scale.data")

Brain_cocaine_DE_stim_C27 <- FindMarkers(Brain.integrated,ident.1 = "27_cocaine",ident.2 = "27_sucrose",test.use = "MAST", assay = "SCT", slot = "scale.data")

Brain_cocaine_DE_stim_C28 <- FindMarkers(Brain.integrated,ident.1 = "28_cocaine",ident.2 = "28_sucrose",test.use = "MAST", assay = "SCT", slot = "scale.data")

Brain_cocaine_DE_stim_C29 <- FindMarkers(Brain.integrated,ident.1 = "29_cocaine",ident.2 = "29_sucrose",test.use = "MAST", assay = "SCT", slot = "scale.data")

Brain_cocaine_DE_stim_C30 <- FindMarkers(Brain.integrated,ident.1 = "30_cocaine",ident.2 = "30_sucrose",test.use = "MAST", assay = "SCT", slot = "scale.data")

Brain_cocaine_DE_stim_C31 <- FindMarkers(Brain.integrated,ident.1 = "31_cocaine",ident.2 = "31_sucrose",test.use = "MAST", assay = "SCT", slot = "scale.data")

Brain_cocaine_DE_stim_C32 <- FindMarkers(Brain.integrated,ident.1 = "32_cocaine",ident.2 = "32_sucrose",test.use = "MAST", assay = "SCT", slot = "scale.data")

Brain_cocaine_DE_stim_C33 <- FindMarkers(Brain.integrated,ident.1 = "33_cocaine",ident.2 = "33_sucrose",test.use = "MAST", assay = "SCT", slot = "scale.data")

Brain_cocaine_DE_stim_C34 <- FindMarkers(Brain.integrated,ident.1 = "34_cocaine",ident.2 = "34_sucrose",test.use = "MAST", assay = "SCT", slot = "scale.data")

Brain_cocaine_DE_stim_C35 <- FindMarkers(Brain.integrated,ident.1 = "35_cocaine",ident.2 = "35_sucrose",test.use = "MAST", assay = "SCT", slot = "scale.data")

##Export results to excel

##names first

write.xlsx(Brain.integrated@assays$RNA@counts@Dimnames[[1]],file="Gene_names.xlsx")

Female_DE_list <- list("Female_C0" = Brain_cocaine_DE_female_stim_C0,"Female_C1"=Brain_cocaine_DE_female_stim_C1,"Female_C2"=Brain_cocaine_DE_female_stim_C2,"Female_C3"=Brain_cocaine_DE_female_stim_C3,"Female_C4"=Brain_cocaine_DE_female_stim_C4,"Female_C5"=Brain_cocaine_DE_female_stim_C5,"Female_C6"=Brain_cocaine_DE_female_stim_C6,"Female_C7"=Brain_cocaine_DE_female_stim_C7,"Female_C8"=Brain_cocaine_DE_female_stim_C8,"Female_C9"=Brain_cocaine_DE_female_stim_C9,"Female_C10"=Brain_cocaine_DE_female_stim_C10,"Female_C11"=Brain_cocaine_DE_female_stim_C11,"Female_C12"=Brain_cocaine_DE_female_stim_C12,"Female_C13"=Brain_cocaine_DE_female_stim_C13,"Female_C14"=Brain_cocaine_DE_female_stim_C14,"Female_15"=Brain_cocaine_DE_female_stim_C15,"Female_C16"=Brain_cocaine_DE_female_stim_C16,"Female_C17"=Brain_cocaine_DE_female_stim_C17,"Female_C18"=Brain_cocaine_DE_female_stim_C18,"Female_C19"=Brain_cocaine_DE_female_stim_C19,"Female_C20"=Brain_cocaine_DE_female_stim_C20,"Female_C21"=Brain_cocaine_DE_female_stim_C21,"Female_C22"=Brain_cocaine_DE_female_stim_C22,"Female_C23"=Brain_cocaine_DE_female_stim_C23,"Female_C24"=Brain_cocaine_DE_female_stim_C24,"Female_C25"=Brain_cocaine_DE_female_stim_C25,"Female_C26"=Brain_cocaine_DE_female_stim_C26,"Female_C27"=Brain_cocaine_DE_female_stim_C27,"Female_C28"=Brain_cocaine_DE_female_stim_C28,"Female_C29"=Brain_cocaine_DE_female_stim_C29,"Female_C30"=Brain_cocaine_DE_female_stim_C30,"Female_C31"=Brain_cocaine_DE_female_stim_C31,"Female_C32"=Brain_cocaine_DE_female_stim_C32,"Female_C33"=Brain_cocaine_DE_female_stim_C33,"Female_C34"=Brain_cocaine_DE_female_stim_C34,"Female_C35"=Brain_cocaine_DE_female_stim_C35)

write.xlsx(Female_DE_list,file="Brain_Cocaine_Female_DE.xlsx",rowNames=TRUE)

Male_DE_list <- list("Male_C0" = Brain_cocaine_DE_male_stim_C0,"Male_C1"=Brain_cocaine_DE_male_stim_C1,"Male_C2"=Brain_cocaine_DE_male_stim_C2,"Male_C3"=Brain_cocaine_DE_male_stim_C3,"Male_C4"=Brain_cocaine_DE_male_stim_C4,"Male_C5"=Brain_cocaine_DE_male_stim_C5,"Male_C6"=Brain_cocaine_DE_male_stim_C6,"Male_C7"=Brain_cocaine_DE_male_stim_C7,"Male_C8"=Brain_cocaine_DE_male_stim_C8,"Male_C9"=Brain_cocaine_DE_male_stim_C9,"Male_C10"=Brain_cocaine_DE_male_stim_C10,"Male_C11"=Brain_cocaine_DE_male_stim_C11,"Male_C12"=Brain_cocaine_DE_male_stim_C12,"Male_C13"=Brain_cocaine_DE_male_stim_C13,"Male_C14"=Brain_cocaine_DE_male_stim_C14,"Male_C15"=Brain_cocaine_DE_male_stim_C15,"Male_C16"=Brain_cocaine_DE_male_stim_C16,"Male_C17"=Brain_cocaine_DE_male_stim_C17,"Male_C18"=Brain_cocaine_DE_male_stim_C18,"Male_C19"=Brain_cocaine_DE_male_stim_C19,"Male_C20"=Brain_cocaine_DE_male_stim_C20,"Male_C21"=Brain_cocaine_DE_male_stim_C21,"Male_C22"=Brain_cocaine_DE_male_stim_C22,"Male_C23"=Brain_cocaine_DE_male_stim_C23,"Male_C24"=Brain_cocaine_DE_male_stim_C24,"Male_C25"=Brain_cocaine_DE_male_stim_C25,"Male_C26"=Brain_cocaine_DE_male_stim_C26,"Male_C27"=Brain_cocaine_DE_male_stim_C27,"Male_C28"=Brain_cocaine_DE_male_stim_C28,"Male_C29"=Brain_cocaine_DE_male_stim_C29,"Male_C30"=Brain_cocaine_DE_male_stim_C30,"Male_C31"=Brain_cocaine_DE_male_stim_C31,"Male_C32"=Brain_cocaine_DE_male_stim_C32,"Male_C33"=Brain_cocaine_DE_male_stim_C33,"Male_C34"=Brain_cocaine_DE_male_stim_C34,"Male_C35"=Brain_cocaine_DE_male_stim_C35)

write.xlsx(Male_DE_list,file="Brain_Cocaine_Male_DE.xlsx",rowNames=TRUE)

Global_DE_list <- list("Global_C0" = Brain_cocaine_DE_stim_C0,"Global_C1"=Brain_cocaine_DE_stim_C1,"Global_C2"=Brain_cocaine_DE_stim_C2,"Global_C3"=Brain_cocaine_DE_stim_C3,"Global_C4"=Brain_cocaine_DE_stim_C4,"Global_C5"=Brain_cocaine_DE_stim_C5,"Global_C6"=Brain_cocaine_DE_stim_C6,"Global_C7"=Brain_cocaine_DE_stim_C7,"Global_C8"=Brain_cocaine_DE_stim_C8,"Global_C9"=Brain_cocaine_DE_stim_C9,"Global_C10"=Brain_cocaine_DE_stim_C10,"Global_C11"=Brain_cocaine_DE_stim_C11,"Global_C12"=Brain_cocaine_DE_stim_C12,"Global_C13"=Brain_cocaine_DE_stim_C13,"Global_C14"=Brain_cocaine_DE_stim_C14,"Global_C15"=Brain_cocaine_DE_stim_C15,"Global_C16"=Brain_cocaine_DE_stim_C16,"Global_C17"=Brain_cocaine_DE_stim_C17,"Global_C18"=Brain_cocaine_DE_stim_C18,"Global_C19"=Brain_cocaine_DE_stim_C19,"Global_C20"=Brain_cocaine_DE_stim_C20,"Global_C21"=Brain_cocaine_DE_stim_C21,"Global_C22"=Brain_cocaine_DE_stim_C22,"Global_C23"=Brain_cocaine_DE_stim_C23,"Global_C24"=Brain_cocaine_DE_stim_C24,"Global_C25"=Brain_cocaine_DE_stim_C25,"Global_C26"=Brain_cocaine_DE_stim_C26,"Global_C27"=Brain_cocaine_DE_stim_C27,"Global_C28"=Brain_cocaine_DE_stim_C28,"Global_C29"=Brain_cocaine_DE_stim_C29,"Global_C30"=Brain_cocaine_DE_stim_C30,"Global_C31"=Brain_cocaine_DE_stim_C31,"Global_C32"=Brain_cocaine_DE_stim_C32,"Global_C33"=Brain_cocaine_DE_stim_C33,"Global_C34"=Brain_cocaine_DE_stim_C34,"Global_C35"=Brain_cocaine_DE_stim_C35)

write.xlsx(Global_DE_list,file="Brain_Cocaine_Global_DE.xlsx",rowNames=TRUE)

##Shared venn part (numbers derived from Supplementary table S9)

shared_c0 <- Venn(SetNames=c("Female","Male"),Weight = c(`10`= 41, `01` = 86, `11` = 48))

gridExtra::grid.arrange(grid::grid.grabExpr(plot(shared_c0, doWeights = TRUE, type = "circles")), top=textGrob("Cluster 0",gp=gpar(fontsize=24,font=8)))

##Global analysis to assess consistent responses across clusters

Brain_cocaine_DE_rankorder_upreg <- read_excel("Brain_Cocaine_Global_rank_order.xlsx",sheet="Upreg_rankorder")

Brain_cocaine_DE_rankorder_downreg <- read_excel("Brain_Cocaine_Global_rank_order.xlsx",sheet="Downreg_rankorder")

Brain_cocaine_DE_rankorder_upreg <- as.data.frame(Brain_cocaine_DE_rankorder_upreg)

Brain_cocaine_DE_rankorder_downreg <- as.data.frame(Brain_cocaine_DE_rankorder_downreg)

Brain_cocaine_DE_upreg_topk <- j0.multi(Brain_cocaine_DE_rankorder_upreg,d=6,v=5)

sapply(Brain_cocaine_DE_upreg_topk,head)

upreg_k <- Brain_cocaine_DE_upreg_topk$maxK

upreg_C0 <- as.character(Brain_cocaine_DE_rankorder_upreg[1:upreg_k,1])

upreg_C1 <- as.character(Brain_cocaine_DE_rankorder_upreg[1:upreg_k,2])

upreg_C2 <- as.character(Brain_cocaine_DE_rankorder_upreg[1:upreg_k,3])

upreg_C3 <- as.character(Brain_cocaine_DE_rankorder_upreg[1:upreg_k,4])

upreg_C4 <- as.character(Brain_cocaine_DE_rankorder_upreg[1:upreg_k,5])

upreg_C5 <- as.character(Brain_cocaine_DE_rankorder_upreg[1:upreg_k,6])

upreg_C6 <- as.character(Brain_cocaine_DE_rankorder_upreg[1:upreg_k,7])

upreg_C7 <- as.character(Brain_cocaine_DE_rankorder_upreg[1:upreg_k,8])

upreg_C8 <- as.character(Brain_cocaine_DE_rankorder_upreg[1:upreg_k,9])

upreg_C9 <- as.character(Brain_cocaine_DE_rankorder_upreg[1:upreg_k,10])

upreg_C10 <- as.character(Brain_cocaine_DE_rankorder_upreg[1:upreg_k,11])

upreg_C11 <- as.character(Brain_cocaine_DE_rankorder_upreg[1:upreg_k,12])

upreg_C12 <- as.character(Brain_cocaine_DE_rankorder_upreg[1:upreg_k,13])

upreg_C13 <- as.character(Brain_cocaine_DE_rankorder_upreg[1:upreg_k,14])

upreg_C14 <- as.character(Brain_cocaine_DE_rankorder_upreg[1:upreg_k,15])

upreg_C15 <- as.character(Brain_cocaine_DE_rankorder_upreg[1:upreg_k,16])

upreg_C16 <- as.character(Brain_cocaine_DE_rankorder_upreg[1:upreg_k,17])

upreg_C17 <- as.character(Brain_cocaine_DE_rankorder_upreg[1:upreg_k,18])

upreg_C18 <- as.character(Brain_cocaine_DE_rankorder_upreg[1:upreg_k,19])

upreg_C19 <- as.character(Brain_cocaine_DE_rankorder_upreg[1:upreg_k,20])

upreg_C20 <- as.character(Brain_cocaine_DE_rankorder_upreg[1:upreg_k,21])

upreg_C21 <- as.character(Brain_cocaine_DE_rankorder_upreg[1:upreg_k,22])

upreg_C22 <- as.character(Brain_cocaine_DE_rankorder_upreg[1:upreg_k,23])

upreg_C23 <- as.character(Brain_cocaine_DE_rankorder_upreg[1:upreg_k,24])

upreg_C24 <- as.character(Brain_cocaine_DE_rankorder_upreg[1:upreg_k,25])

upreg_C25 <- as.character(Brain_cocaine_DE_rankorder_upreg[1:upreg_k,26])

upreg_C26 <- as.character(Brain_cocaine_DE_rankorder_upreg[1:upreg_k,27])

upreg_C27 <- as.character(Brain_cocaine_DE_rankorder_upreg[1:upreg_k,28])

upreg_C28 <- as.character(Brain_cocaine_DE_rankorder_upreg[1:upreg_k,29])

upreg_C29 <- as.character(Brain_cocaine_DE_rankorder_upreg[1:upreg_k,30])

upreg_C30 <- as.character(Brain_cocaine_DE_rankorder_upreg[1:upreg_k,31])

upreg_C31 <- as.character(Brain_cocaine_DE_rankorder_upreg[1:upreg_k,32])

upreg_C32 <- as.character(Brain_cocaine_DE_rankorder_upreg[1:upreg_k,33])

upreg_C33 <- as.character(Brain_cocaine_DE_rankorder_upreg[1:upreg_k,34])

upreg_C34 <- as.character(Brain_cocaine_DE_rankorder_upreg[1:upreg_k,35])

upreg_C35 <- as.character(Brain_cocaine_DE_rankorder_upreg[1:upreg_k,36])

upreg_list <- list(upreg_C0,upreg_C1,upreg_C10,upreg_C11,upreg_C12,upreg_C13,upreg_C14,upreg_C15,upreg_C16,upreg_C17,upreg_C18,upreg_C19,upreg_C2,upreg_C20,upreg_C21,upreg_C22,upreg_C23,upreg_C24,upreg_C25,upreg_C26,upreg_C27,upreg_C28,upreg_C29,upreg_C3,upreg_C30,upreg_C31,upreg_C32,upreg_C33,upreg_C34,upreg_C35,upreg_C4,upreg_C5,upreg_C6,upreg_C7,upreg_C8,upreg_C9)

upreg_common <- unique(unlist(upreg_list))

upreg_space = list(upreg_common,upreg_common,upreg_common,upreg_common,upreg_common,upreg_common,upreg_common,upreg_common,upreg_common,upreg_common,upreg_common,upreg_common,upreg_common,upreg_common,upreg_common,upreg_common,upreg_common,upreg_common,upreg_common,upreg_common,upreg_common,upreg_common,upreg_common,upreg_common,upreg_common,upreg_common,upreg_common,upreg_common,upreg_common,upreg_common,upreg_common,upreg_common,upreg_common,upreg_common,upreg_common,upreg_common)

upreg_out_Borda=Borda(upreg_list,upreg_space)

upreg_out_MC=MC(upreg_list,upreg_space)

upreg_out_CEMC=CEMC(upreg_list,upreg_space,N=2000)

upreg_agg=list(ARM=upreg_out_Borda$TopK[,1],MED=upreg_out_Borda$TopK[,2],GEO=upreg_out_Borda$TopK[,3],L2N=upreg_out_Borda$TopK[,4,],MC1=upreg_out_MC$MC1.TopK,MC2=upreg_out_MC$MC2.TopK,MC3=upreg_out_MC$MC3.TopK,CEMC=upreg_out_CEMC$TopK)

Kendall.plot(upreg_list,upreg_agg,upreg_space,algorithm=c("ARM","MED","GEO","L2N","MC1","MC2","MC3","CEMC"))

Brain_cocaine_DE_downreg_topk <- j0.multi(Brain_cocaine_DE_rankorder_downreg,d=6,v=5)

sapply(Brain_cocaine_DE_downreg_topk,head)

downreg_k <- Brain_cocaine_DE_downreg_topk$maxK

downreg_C0 <- as.character(Brain_cocaine_DE_rankorder_downreg[1:downreg_k,1])

downreg_C1 <- as.character(Brain_cocaine_DE_rankorder_downreg[1:downreg_k,2])

downreg_C2 <- as.character(Brain_cocaine_DE_rankorder_downreg[1:downreg_k,3])

downreg_C3 <- as.character(Brain_cocaine_DE_rankorder_downreg[1:downreg_k,4])

downreg_C4 <- as.character(Brain_cocaine_DE_rankorder_downreg[1:downreg_k,5])

downreg_C5 <- as.character(Brain_cocaine_DE_rankorder_downreg[1:downreg_k,6])

downreg_C6 <- as.character(Brain_cocaine_DE_rankorder_downreg[1:downreg_k,7])

downreg_C7 <- as.character(Brain_cocaine_DE_rankorder_downreg[1:downreg_k,8])

downreg_C8 <- as.character(Brain_cocaine_DE_rankorder_downreg[1:downreg_k,9])

downreg_C9 <- as.character(Brain_cocaine_DE_rankorder_downreg[1:downreg_k,10])

downreg_C10 <- as.character(Brain_cocaine_DE_rankorder_downreg[1:downreg_k,11])

downreg_C11 <- as.character(Brain_cocaine_DE_rankorder_downreg[1:downreg_k,12])

downreg_C12 <- as.character(Brain_cocaine_DE_rankorder_downreg[1:downreg_k,13])

downreg_C13 <- as.character(Brain_cocaine_DE_rankorder_downreg[1:downreg_k,14])

downreg_C14 <- as.character(Brain_cocaine_DE_rankorder_downreg[1:downreg_k,15])

downreg_C15 <- as.character(Brain_cocaine_DE_rankorder_downreg[1:downreg_k,16])

downreg_C16 <- as.character(Brain_cocaine_DE_rankorder_downreg[1:downreg_k,17])

downreg_C17 <- as.character(Brain_cocaine_DE_rankorder_downreg[1:downreg_k,18])

downreg_C18 <- as.character(Brain_cocaine_DE_rankorder_downreg[1:downreg_k,19])

downreg_C19 <- as.character(Brain_cocaine_DE_rankorder_downreg[1:downreg_k,20])

downreg_C20 <- as.character(Brain_cocaine_DE_rankorder_downreg[1:downreg_k,21])

downreg_C21 <- as.character(Brain_cocaine_DE_rankorder_downreg[1:downreg_k,22])

downreg_C22 <- as.character(Brain_cocaine_DE_rankorder_downreg[1:downreg_k,23])

downreg_C23 <- as.character(Brain_cocaine_DE_rankorder_downreg[1:downreg_k,24])

downreg_C24 <- as.character(Brain_cocaine_DE_rankorder_downreg[1:downreg_k,25])

downreg_C25 <- as.character(Brain_cocaine_DE_rankorder_downreg[1:downreg_k,26])

downreg_C26 <- as.character(Brain_cocaine_DE_rankorder_downreg[1:downreg_k,27])

downreg_C27 <- as.character(Brain_cocaine_DE_rankorder_downreg[1:downreg_k,28])

downreg_C28 <- as.character(Brain_cocaine_DE_rankorder_downreg[1:downreg_k,29])

downreg_C29 <- as.character(Brain_cocaine_DE_rankorder_downreg[1:downreg_k,30])

downreg_C30 <- as.character(Brain_cocaine_DE_rankorder_downreg[1:downreg_k,31])

downreg_C31 <- as.character(Brain_cocaine_DE_rankorder_downreg[1:downreg_k,32])

downreg_C32 <- as.character(Brain_cocaine_DE_rankorder_downreg[1:downreg_k,33])

downreg_C33 <- as.character(Brain_cocaine_DE_rankorder_downreg[1:downreg_k,34])

downreg_C34 <- as.character(Brain_cocaine_DE_rankorder_downreg[1:downreg_k,35])

downreg_C35 <- as.character(Brain_cocaine_DE_rankorder_downreg[1:downreg_k,36])

downreg_list <- list(downreg_C0,downreg_C1,downreg_C10,downreg_C11,downreg_C12,downreg_C13,downreg_C14,downreg_C15,downreg_C16,downreg_C17,downreg_C18,downreg_C19,downreg_C2,downreg_C20,downreg_C21,downreg_C22,downreg_C23,downreg_C24,downreg_C25,downreg_C26,downreg_C27,downreg_C28,downreg_C29,downreg_C3,downreg_C30,downreg_C31,downreg_C32,downreg_C33,downreg_C34,downreg_C35,downreg_C4,downreg_C5,downreg_C6,downreg_C7,downreg_C8,downreg_C9)

downreg_common <- unique(unlist(downreg_list))

downreg_space = list(downreg_common,downreg_common,downreg_common,downreg_common,downreg_common,downreg_common,downreg_common,downreg_common,downreg_common,downreg_common,downreg_common,downreg_common,downreg_common,downreg_common,downreg_common,downreg_common,downreg_common,downreg_common,downreg_common,downreg_common,downreg_common,downreg_common,downreg_common,downreg_common,downreg_common,downreg_common,downreg_common,downreg_common,downreg_common,downreg_common,downreg_common,downreg_common,downreg_common,downreg_common,downreg_common,downreg_common)

downreg_out_Borda=Borda(downreg_list,downreg_space)

downreg_out_MC=MC(downreg_list,downreg_space)

downreg_out_CEMC=CEMC(downreg_list,downreg_space,N=2000)

downreg_agg=list(ARM=downreg_out_Borda$TopK[,1],MED=downreg_out_Borda$TopK[,2],GEO=downreg_out_Borda$TopK[,3],L2N=downreg_out_Borda$TopK[,4,],MC1=downreg_out_MC$MC1.TopK,MC2=downreg_out_MC$MC2.TopK,MC3=downreg_out_MC$MC3.TopK,CEMC=downreg_out_CEMC$TopK)

Kendall.plot(downreg_list,downreg_agg,downreg_space,algorithm=c("ARM","MED","GEO","L2N","MC1","MC2","MC3","CEMC"))

##Globally regulated genes - visualization

DefaultAssay(Brain.integrated)<-"SCT"

FeaturePlot(Brain.integrated,features="Dmel-CR34335",split.by = "gender_stim",cols=c("grey","purple"),max.cutoff=2,min.cutoff = 1.99,ncol=2)

FeaturePlot(Brain.integrated,features="Dmel-CG11352",split.by = "gender_stim",cols=c("grey","red"),max.cutoff=2,min.cutoff = 1.99,ncol=2)

FeaturePlot(Brain.integrated,features="Dmel-CG17228",split.by = "gender_stim",cols=c("grey","blue"),max.cutoff=2.5,min.cutoff = 1.99,ncol=2)

FeaturePlot(Brain.integrated,features="Dmel-CG31795",split.by = "gender_stim",cols=c("grey","greenyellow"),max.cutoff=4,min.cutoff = 2,ncol=2)

FeaturePlot(Brain.integrated,features="Dmel-CG32169",split.by = "gender_stim",cols=c("grey","cyan2"),max.cutoff=3,min.cutoff = 1.5,ncol=2)

FeaturePlot(Brain.integrated,features="Dmel-CG30425",split.by = "gender_stim",cols=c("grey","maroon1"),max.cutoff=2,ncol=2)

FeaturePlot(Brain.integrated,features="Dmel-CR34094",split.by = "gender_stim",cols=c("grey","dark orange"),max.cutoff=2.5,ncol=2)

FeaturePlot(Brain.integrated,features="Dmel-CG4899",split.by = "gender_stim",cols=c("grey","blue"),max.cutoff=0.2,ncol=2)

## Highlight interesting clusters

DimPlot(Brain.integrated,reduction="umap",group.by="Celltype",split.by = "gender_stim",cells.highlight = WhichCells(Brain.integrated,idents=c("22_female_cocaine","22_female_sucrose","22_male_cocaine","22_male_sucrose")),cols.highlight = "purple",sizes.highlight = 0.25) + NoLegend()

##Expression pattern for Interesting genes

FeaturePlot(Brain.integrated,features="Dmel-CG3822",split.by = "gender_stim",cols=c("grey","purple"),max.cutoff=1,min.cutoff = 0.5,ncol=2)

FeaturePlot(Brain.integrated,features="Dmel-CG3822",split.by = "gender_stim",cols=c("grey","purple"),max.cutoff=1,min.cutoff = 0.5,ncol=2)

FeaturePlot(Brain.integrated,features="Dmel-CR33925",split.by = "gender_stim",cols=c("grey","maroon1"),max.cutoff=1.5,min.cutoff = 0.75,ncol=2)

FeaturePlot(Brain.integrated,features="Dmel-CR31400",split.by = "gender_stim",cols=c("grey","cyan2"),max.cutoff=3.5,min.cutoff = 2.75,ncol=2)

FeaturePlot(Brain.integrated,features="Dmel-CG10693",split.by = "gender_stim",cols=c("grey","green"),max.cutoff=2.5,min.cutoff = 1.85,ncol=2)

FeaturePlot(Brain.integrated,features="Dmel-CG2239",split.by = "gender_stim",cols=c("grey","blue"),max.cutoff=3,min.cutoff = 1.85,ncol=2)

FeaturePlot(Brain.integrated,features="Dmel-CG33527",split.by = "gender_stim",cols=c("grey","red"),max.cutoff=0.75,min.cutoff = 0.1,ncol=2)

FeaturePlot(Brain.integrated,features="Dmel-CG18345",split.by = "gender_stim",cols=c("grey","steelblue1"),max.cutoff=1.5,min.cutoff = 0.5,ncol=2)

FeaturePlot(Brain.integrated,features="Dmel-CG33517",split.by = "gender_stim",cols=c("grey","royalblue1"),max.cutoff=2.75,min.cutoff = 2.5,ncol=2)

FeaturePlot(Brain.integrated,features="Dmel-CG6702",split.by = "gender_stim",cols=c("grey","magenta"),max.cutoff=2.75,min.cutoff = 2.5,ncol=2)

FeaturePlot(Brain.integrated,features="Dmel-CG45477",split.by = "gender_stim",cols=c("grey","dark green"),max.cutoff=3,min.cutoff = 2.5,ncol=2)

FeaturePlot(Brain.integrated,features="Dmel-CG14989",split.by = "gender_stim",cols=c("grey","violetred"),max.cutoff=4.5,min.cutoff = 3.75,ncol=2)

FeaturePlot(Brain.integrated,features="Dmel-CG10804",split.by = "gender_stim",cols=c("grey","tomato1"),max.cutoff=1.5,min.cutoff = 0.5,ncol=2)

FeaturePlot(Brain.integrated,features="Dmel-CG4128",split.by = "gender_stim",cols=c("grey","hotpink1"),max.cutoff=2.0,min.cutoff = 1.5,ncol=2)

FeaturePlot(Brain.integrated,features="Dmel-CG11387",split.by = "gender_stim",cols=c("grey","chocolate"),max.cutoff=2.0,min.cutoff = 1.5,ncol=2)

FeaturePlot(Brain.integrated,features="Dmel-CG34418",split.by = "gender_stim",cols=c("grey","darkgoldenrod1"),max.cutoff=2.0,min.cutoff = 1.5,ncol=2)

FeaturePlot(Brain.integrated,features="Dmel-CG34387",split.by = "gender_stim",cols=c("grey","seagreen"),max.cutoff=2.5,min.cutoff = 2,ncol=2)

FeaturePlot(Brain.integrated,features="Dmel-CG31000",split.by = "gender_stim",cols=c("grey","darkorchid"),max.cutoff=2.0,min.cutoff = 1.5,ncol=2)

FeaturePlot(Brain.integrated,features="Dmel-CG7503",split.by = "gender_stim",cols=c("grey","navy"),max.cutoff=2.0,min.cutoff = 1.5,ncol=2)

FeaturePlot(Brain.integrated,features="Dmel-CG32490",split.by = "gender_stim",cols=c("grey","lawngreen"),max.cutoff=2.75,min.cutoff = 2.25,ncol=2)

FeaturePlot(Brain.integrated,features="Dmel-CG1130",split.by = "gender_stim",cols=c("grey","darkmagenta"),max.cutoff=2.35,min.cutoff = 2,ncol=2)

FeaturePlot(Brain.integrated,features="Dmel-CG4838",split.by = "gender_stim",cols=c("grey","limegreen"),max.cutoff=2,min.cutoff = 1.5,ncol=2)

FeaturePlot(Brain.integrated,features="Dmel-CG4838",split.by = "gender_stim",cols=c("grey","purple"),max.cutoff=2,min.cutoff = 1.5,ncol=2)

FeaturePlot(Brain.integrated,features="Dmel-CG32169",split.by = "gender_stim",cols=c("grey","red"),max.cutoff=2.5,min.cutoff = 2,ncol=2)

FeaturePlot(Brain.integrated,features="Dmel-CG16944",split.by = "gender_stim",cols=c("grey","blue"),max.cutoff=2.25,min.cutoff = 1.75,ncol=2)

FeaturePlot(Brain.integrated,features="Dmel-CG42338",split.by = "gender_stim",cols=c("grey","green"),max.cutoff=2.25,min.cutoff = 1.75,ncol=2)

FeaturePlot(Brain.integrated,features="Dmel-CG13739",split.by = "gender_stim",cols=c("grey","cyan2"),max.cutoff=1.75,min.cutoff = 1.25,ncol=2)

FeaturePlot(Brain.integrated,features="Dmel-CG1429",split.by = "gender_stim",cols=c("grey","maroon1"),max.cutoff=1.75,min.cutoff = 1.25,ncol=2)

FeaturePlot(Brain.integrated,features="Dmel-CG7533",split.by = "gender_stim",cols=c("grey","dark orange"),max.cutoff=1.5,min.cutoff = 1,ncol=2)

FeaturePlot(Brain.integrated,features="Dmel-CG6575",split.by = "gender_stim",cols=c("grey","steelblue1"),max.cutoff=1.25,min.cutoff = 0.75,ncol=2)

FeaturePlot(Brain.integrated,features="Dmel-CG9887",split.by = "gender_stim",cols=c("grey","royalblue1"),max.cutoff=1,min.cutoff = 0.5,ncol=2)

##heatmap

Brain_cocaine_DE_female_interesting_markers<-c('Dmel-CR32665',

'Dmel-CR34335',

'Dmel-CR33925',

'Dmel-CG31795',

'Dmel-CR31400',

'Dmel-CG14994',

'Dmel-CG6058',

'Dmel-CG42338',

'Dmel-CG17228',

'Dmel-CG31000',

'Dmel-CG9887',

'Dmel-CG5099',

'Dmel-CR34094',

'Dmel-CG30425',

'Dmel-CG43395',

'Dmel-CG31221',

'Dmel-CG32169',

'Dmel-CG4838',

'Dmel-CG2239',

'Dmel-CG10693',

'Dmel-CG44011',

'Dmel-CG4899',

'Dmel-CG3139',

'Dmel-CG17907',

'Dmel-CG17716',

'Dmel-CG12348',

'Dmel-CG5744',

'Dmel-CG32626',

'Dmel-CG32538',

'Dmel-CG14274',

'Dmel-CG43795',

'Dmel-CG32444',

'Dmel-CG1634',

'Dmel-CG3694',

'Dmel-CG7607',

'Dmel-CG2297',

'Dmel-CG7811',

'Dmel-CG3039',

'Dmel-CG18389',

'Dmel-CG10045',

'Dmel-CG16936',

'Dmel-CG6781',

'Dmel-CG4784',

'Dmel-CG33527',

'Dmel-CG3578',

'Dmel-CG18598',

'Dmel-CG3504',

'Dmel-CG4550',

'Dmel-CG3966',

'Dmel-CG5125',

'Dmel-CG18345',

'Dmel-CG3151',

'Dmel-CG7220',

'Dmel-CG7875',

'Dmel-CG3822'

)

DoHeatmap(Brain.integrated,features=Brain_cocaine_DE_female_interesting_markers,group.by="celltype.stim", slot = "scale.data",angle=90,disp.max = 0.5) + NoLegend() + scale_fill_gradientn(colors = c("dark orange","black"))

Idents(Brain.integrated)<-"celltype.gender_stim"

DoHeatmap(Brain.integrated,features=c("Dmel-CR32665","Dmel-CG2239","Dmel-CG10693","Dmel-CG44011"), cells = WhichCells(Brain.integrated,idents=c("11_female_cocaine" , "11_female_sucrose")) , slot = "scale.data",angle=90,disp.max = 0.5) + NoLegend() + scale_fill_gradientn(colors = c("dark orange","black"))

##Obtaining scaled data from SCT for downstream coexpression network for individual cluster using RMTGnet

##example C22 female

C22_cells <-WhichCells(Brain.integrated,ident=22)

Brain_cocaine_DE_female_stim_C22_index <- which(Brain_cocaine_DE_female_stim_C22[,5]<=0.05)

C22_scale_data <- FetchData(Brain.integrated,vars = rownames(Brain_cocaine_DE_female_stim_C22[Brain_cocaine_DE_female_stim_C22_index,]),cells=C22_cells,slot = "scale.data")

##example C22 male

C22_cells <-WhichCells(Brain.integrated,ident=22)

Brain_cocaine_DE_male_stim_C22_index <- which(Brain_cocaine_DE_male_stim_C22[,5]<=0.05)

C22_scale_data <- FetchData(Brain.integrated,vars = rownames(Brain_cocaine_DE_male_stim_C22[Brain_cocaine_DE_male_stim_C22_index,]),cells=C22_cells,slot = "scale.data")

write.xlsx(C22_scale_data,"C22_scale_data.xlsx",rowNames=TRUE)

##example C16 male

C16_cells <-WhichCells(Brain.integrated,ident=16)

Brain_cocaine_DE_male_stim_C16_index <- which(Brain_cocaine_DE_male_stim_C16[,5]<=0.05)

C16_scale_data <- FetchData(Brain.integrated,vars = rownames(Brain_cocaine_DE_male_stim_C16[Brain_cocaine_DE_male_stim_C16_index,]),cells=C16_cells,slot = "scale.data")

write.xlsx(C16_scale_data,"C16_scale_data.xlsx",rowNames=TRUE)

##One can also pull out sample specific data, instead of the entire set. To do this, identify what the # that follows each cells barcode represents. This can be accomplished by setting the idents of the integrated object to sample id (see sections in the beginning) and using WhichCells and giving it the sample ID. It will pull all of the cells that correspond to that sample ID. Look at the _# at the end of the cell barcodes and map the numbers to specific samples. In this dataset the numbers ranged from 1 to 8 because there are 8 total samples. Parse out the exported csv (or .txt) file to only contain cells from samples of interest.
