# Supplementary material for: The Drosophila brain on cocaine at single-cell resolution
Source: Genome Res. 2021 Oct;31(10):1927–37. doi: 10.1101/gr.268037.120 (PMC8494231; doi:10.1101/gr.268037.120)
Supplement: Supplemental Material [file supp_gr.268037.120_Supplemental_Material.pdf]

Supplemental Material

The *Drosophila* brain on cocaine at single cell resolution

Brandon M. Baker*, Sneha S. Mokashi*, Vijay Shankar*, Jeffrey S. Hatfield, Rachel C. Hannah, Trudy F. C. Mackay# and Robert R. H. Anholt#

Center for Human Genetics, Department of Genetics and Biochemistry, Clemson University, Greenwood, SC29646, USA

* These authors contributed equally

TFCM and RRHA are joint corresponding authors

Corresponding authors: Robert R. H. Anholt, ranholt@clemson.edu (tel. 864-889-0521)

Trudy F. C. Mackay, tmackay@clemson.edu (tel. 864-889-0522)

Table of Contents

Supplemental Methods:

Drosophila stock……………………………………………………………………………..4

Cocaine exposure……………………………………………………………………4-5

Behaviors……….………..……………………………………………………….............5-6

Brain dissection and dissociation…...……………………………………………………...6

Library preparation and sequencing……………………………………………………...7

FASTQ generation, demultiplexing and alignment………………………………………7

Preprocessing, integration and cell-type clustering…………………………….……….7-8

Differential expression…………………………………………………………………......8

Simulation of bulk RNAseq response………………………………………………………8

Cluster-specific co-expression networks……………………………...………………..9

Supplemental Tables

Table_S1: Raw behavioral data of flies exposed to cocaine………………………………10

Table_S2: Summary of sequencing and alignment statistics. ……………………………..11

Table_S3: Differentially expressed genes after cocaine exposure of males and females and their top human orthologs. ……..………………………………………………………..12

Table_S4: Characterization of cell clusters through combinations of biomarkers……13-14

Table_S5: Genes that were consistently upregulated and downregulated across multiple clusters due to acute cocaine exposure..…………………….………………………….....15

Table_S6: Results from differential expression analysis for each cluster from the male and female datasets combined.……………………….…………………………………....15

Table_S7: Results from differential expression analysis for each cluster from the male dataset.……...………………….………………………………….………………….….…..15

Table_S8: Results from differential expression analysis for each cluster from the female dataset…………………….……………………………………….……………………………15

Table_S9: Quantification of shared and unique differentially expressed genes due to exposure to cocaine between the males and females within each cluster.………………..15

Table_S10: Functional enrichment analyses of differentially expressed genes in selected clusters due to cocaine exposure……………….……………………………………………....15

Supplemental Figures:

Figure S1: Visualization of gene expression patterns using UMAP projections…...……16

Figure S2: UMAP visualization of expression patterns for genes that respond globally to cocaine exposure..…………………….……………….………………….………..…………..…17-18

Figure S3: Full co-expression network analysis and TOM plot of DEGs from the male C16 cluster……………………….……………………...…………..…………………….……..19

Figure S4: MCODE Subnetworks from co-expression network analysis and TOM plot of DEGs from the male C22 cluster………………….…………………….…………………...20

Supplemental Movies

Movie S1: Startle response assay………………….……………………………………..21

Movie S2: Grooming behavior of a male exposed to cocaine……………………………...21

Movie S3: Seizure of a male exposed to cocaine……………………………………………21

Supplemental References………………………...……………………………………...22-23

Supplemental Methods

Drosophila stock

Canton S (B) flies (Norga et al. 2003) were maintained on standard cornmeal/yeast/molasses-agar culture medium at 25°C on a 12:12 hour light:dark cycle with 50% humidity in controlled adult density vials to prevent overcrowding. Briefly, 5 males and 5 females were placed into a vial and allowed to mate for two days before being cleared. Progeny from these vials were collected after eclosion and aged for 3-5 days before experimentation.

Cocaine exposure

Cocaine.HCl was obtained from the National Institute on Drug Abuse under Drug Enforcement Administration license RA0443159. To expose flies to cocaine, we performed a modified version of the capillary feeder (CAFÉ) assay (Ja et al. 2007). We collected 200 Canton S (B) flies between 3 and 5 days old using CO_2 anesthesia, sexes separately. We placed them individually in culture vials containing cornmeal/yeast/molasses/agar culture medium (Genesee Scientific, Inc., San Diego, CA) and allowed them to recover for 24 hours before experimentation. Between 3:00-5:00 PM on the day before the assay, we transferred the flies to vials containing 1.5% agar (Sigma Aldrich, St. Louis, MO) in which a capillary (VWR International, Radnor, PA: 12.7 cm long, 5 μl total volume) filled completely with a solution of 4% sucrose (Sigma Aldrich) and 1% yeast (Fisher Scientific, Hampton, NH) was inserted. The next morning, we replaced the sucrose capillaries for 100 flies with capillaries containing 4% sucrose supplemented with 1 μg/μL of cocaine and 1% yeast; for the other 100 flies, we replaced the sucrose capillaries with fresh 4% sucrose and 1% yeast with no drug. A droplet of mineral oil (Sigma Aldrich) was added to the top of each capillary to minimize evaporation. We collected the first 40 flies that consumed 0.53 μL of cocaine and the first 40 flies that consumed 0.53 μL of sucrose, corresponding to an 8 mm reduction in the height

of the solution in the capillary. All experiments were carried out between 8 AM and 11 AM. Flies were allowed to feed for no more than 2 hours.

Behaviors

We measured negative geotaxis and startle response of individual flies within a 10-minute time-frame immediately following acute exposure to cocaine in the CAFÉ assay. We quantified grooming and seizures in addition to measuring the behavioral response in each assay. Excessive grooming was defined as more than 10 seconds of constant grooming (Video S2). Seizure activity was defined as severe muscle tremors that prevented the fly from moving normally (Video S3).

Negative geotaxis: Following acute cocaine consumption, we placed each fly in a 14.8 cm-tall clear glass vial with its circumference marked 7.5 cm up the vial. Flies were given 30 seconds to acclimate to the vials. We then tapped the flies to the bottom of the vial and recorded the time taken for each fly to cross the 7.5 cm mark, with a maximum allowed climb time of 30 seconds. Flies that did not pass the mark within 30 seconds were designated as “did not finish”. The numbers of flies tested are indicated in the legend to Figure 1. Significant differences from control were assessed using one-tailed Student’s *t*-test. Grooming and seizure activity were also scored at this time and differences between control flies and flies exposed to cocaine were assessed using Fisher’s exact test.

Startle response: Following acute cocaine consumption, we tested single flies in their vials for acute startle response. To ensure the same amount of mechanical stimulation for all trials, we constructed a ‘fly drop tower’ in which all vials were dropped 42 cm and then secured in a horizontal position. As soon as the vials attained a horizontal position the flies were observed for 45 seconds and the total time each fly spent moving was recorded (Video S1). The numbers of flies tested are indicated in the legend to Figure 1. Significant differences from control were

assessed using one-tailed Student's *t*-test. Grooming and seizure activity were also scored at this time and differences between control flies and flies exposed to cocaine were assessed using Fisher's exact test. While grooming, flies were stationary.

Brain dissection and dissociation

Brains were dissected from each fly immediately after it consumed the designated amount of sucrose or cocaine solution. We used a dissociation protocol modified from Croset et al. (2018) and Davie et al. (2018). We dissected brains in cold D-PBS (Gibco, Thermo Fisher Scientific, Waltham, MA) and collected them into 1.7 ml tubes in cold Schneider's medium (Gibco). We collected 20 brains per sample within one hour. We collected eight samples of 20 brains from males and females exposed to cocaine or sucrose, with two biological replicates per treatment and sex. We replaced the D-PBS in the dissection dish after dissecting 2 brains to ensure that it stayed cold and we used separate drops of buffer for decapitation and brain dissection to avoid contaminating the brain samples. We centrifuged the samplesn at 300xg at 4°C for 5 min and removed the supernatant. We then added 450µl of collagenase solution (50 ul of fresh 25mg/ml collagenase (Gibco) in sterile water + 400µl of Schneider's medium), flicked the tube gently and allowed the brains to incubate at room temperature for 30 min. We replaced the collagenase solution after centrifugation with PBS + 0.04% BSA (NEB, Ipswich, MA). We mechanically dissociated the brains slowly and gently, using stepwise trituration - P200 pipette 5 times, 23G needle pre-wetted with PBS + BSA 5 times, and 27G pre-wetted needle 5 times. We passed the suspension through a pre-wetted 10µm strainer (Celltrics, Görlitz, Germany) aided by gentle tapping. We added 50ul of PBS+BSA to aid flow of the suspension through the strainer. We counted live cells using a hemocytometer with trypan blue exclusion. We proceeded with GEM generation using the Chromium controller (10X Genomics, Pleasanton, CA) if we had a live cell count of > 500 live cells/µl.

Library preparation and sequencing

We made libraries after GEM generation in accordance with 10X Genomics v3.1 protocols. We determined fragment sizes using Agilent Tapestation kits (Agilent, Santa Clara, CA) - d5000 for amplified cDNA and d1000 for libraries. However, the samples were not diluted at either step since these were not high concentration libraries. We measured the concentrations of amplified cDNA and the final libraries using a Qubit 1X dsDNA HS kit, also without dilution. In addition to Qubit, we quantified the final library concentrations using a qPCR based library quantification kit (KAPA Biosystems, Roche, Basel, Switzerland) in order to measure the concentration of fragment sizes of interest in accordance with the manufacturer's recommendations. We used 12 cycles for the cDNA amplification and 14 cycles for indexing PCR. We sequenced the final libraries on an S1 flow cell using a Novaseq (Illumina, Inc., San Diego, CA) according to the manufacturer's instructions.

FASTQ generation, demultiplexing and alignment

The *mkfastq* pipeline within Cell Ranger v3.1 (10X Genomics, Pleasanton, CA) was used to convert BCL files from the sequence run folder to demultiplexed FASTQ files. Release 6 version of the *Drosophila melanogaster* reference *GCA_000001215.4* from NCBI Genbank was indexed using the *mkref* pipeline and used for alignment using the *count* pipeline within Cell Ranger v3.1 with the expected cell count parameter set to 5,000 cells. The sequencing and alignment summary is given in Supplemental Table S2.

Preprocessing, integration and cell-type clustering

Raw expression counts output for each sample from the Cell Ranger pipeline was imported and analyzed using the Seurat v3 package in R (Butler et al. 2018). Genes expressed in less than 5 cells and cells with less than 300 or greater than 2500 RNA features were filtered out. Normalization and subsequent integration were performed using *scTransform* pipeline

(Hafemeister and Satija, 2019). To identify the cell-type clusters within the dataset, unsupervised clustering using the *FindClusters* function and a resolution of 0.8 was used. Cluster marker genes were identified using *FindAllMarker* function (min.pct=0.25, logfc.threshold = 0.5, only.pos = TRUE). The top three genes with positive expression for each cluster were extracted and used for cell-type characterization.

Differential expression

Differential expression was performed for each cluster in two ways: (i) after combining male and female samples together to test for effects of cocaine that are common to both sexes; and (ii) testing for effects of cocaine in males and females separately to identify sexually-dimorphic responses. The Pearson residuals output from *scTransform* pipeline was used as input for differential expression (DE) calculation (Hafemeister and Satija, 2019). The *MAST* algorithm was used as the testing methodology in the *FindMarkers* function (test.use = "MAST", assay = "SCT", slot = "scale.data") for each cluster to calculate DE. Clusters with sufficient number of DEGs were subjected to pathway enrichment analysis using the statistical overrepresentation test using the PantherDB (Thomas et al. 2003) and Reactome databases (Fabregat et al. 2016). Pathways with BH-FDR adjusted *P* values < 0.05 were considered statistically enriched.

Simulation of bulk RNAseq response

The results from DE calculation from the combined dataset were used to determine which genes were consistently upregulated and downregulated, respectively, across all clusters as a result of exposure to cocaine. The top 50 ranked differentially upregulated genes for each cluster and the top 20 ranked differentially downregulated genes for each cluster were input into *TopKLists* R package (Schimek et al. 2015).

Cluster-specific co-expression networks

The scaled data from the *scTransform* pipeline for differentially expressed genes from clusters 16 and 22 were extracted for the male samples. These scaled data were used as input for filtering through Random Matrix Theory (RMT; Gibson et al. 2013). The correlations that passed the filtering process were visualized using Cytoscape version 3.7.2. The MCODE algorithm (Bader et al. 2003) was utilized to identify highly interconnected modules within the larger cluster network. Genetic interaction networks were constructed by converting the gene IDs to gene names/symbols using the FlyBase Consortium's 'Query-by-symbols/ID' tool and calculating interactions between gene products using the stringApp plugin within Cytoscape (Doncheva et al. 2019). To identify specific pathways that are enriched in genes within each of the circular groups, we performed statistical overrepresentation tests on the gene IDs from each group using the PantherDB (Thomas et al. 2003) and Reactome (Fabregat et al. 2018) databases. Pathways with BH-FDR adjusted P values < 0.05 were considered statistically enriched.

Supplemental Tables

Table S1: Raw behavioral data of flies exposed to cocaine. Refer to Supplemental_Table_S1.xlsx

Sequencing Sample ID	Number of Reads	Estimated Number of Cells	Mean Reads per Cell	Median Genes per Cell	Sequencing Saturation	Fraction Reads in Cells	Total Genes Detected	Median UMI Counts per Cell
♀ Sucrose Rep 1	87,765,954	9,072	9,674	772	41.60%	71.50%	11,958	1,347
♀ Sucrose Rep 2	72,264,746	11,693	6,180	807	29.70%	80.90%	12,049	1,476
♂ Sucrose Rep 1	123,911,975	13,193	9,392	769	29.20%	79.50%	12,523	1,378
♂ Sucrose Rep 1	114,946,639	11,033	10,418	799	31.10%	72.00%	12,354	1,440
♀ Cocaine Rep 1	120,400,905	13,072	9,211	733	22.40%	69.70%	12,203	1,304
♀ Cocaine Rep 2	142,640,818	9,367	15,228	1,080	52.80%	83.30%	12,618	2,286
♂ Cocaine Rep 1	78,416,962	10,437	7,513	680	22.00%	68.40%	11,631	1,190
♂ Cocaine Rep 2	162,182,108	11,124	14,579	1,207	21.70%	58.70%	12,464	2,868
Undetermined	49,596,498							
Total	967,684,238	88,991						
Minimum	72,264,746	9,072	6,180	680	21.70%	58.70%	11,631	1,190
Maximum	162,182,108	13,193	15,228	1,207	52.80%	83.30%	12,618	2,868
Ratio	2.24	1.45	2.46	1.78	2.43	1.42	1.08	2.41
Mean	112,816,263	11,124	10,274	856	31.31%	73.00%	12,225	1,661
Standard deviation	31,547,982	1520.21	3156.57	185.07	10.91%	8.02%	332.22	592.64

Table S2: Summary of sequencing and alignment statistics.

Table S3: Differentially expressed genes after cocaine exposure of males and females and their top human orthologs. Refer to Supplemental_Table_S3.xlsx

Cluster	Cluster defining genes	Cluster annotation	References
0	***VGlut***/*CG2269/CG32017*	Glutamatergic neurons	Croset et al., 2018
1	*CG14989/*__*Gad1*__*/CG32017*	GABAergic neurons	Croset et al., 2018
2	***spab/jeb***/*CG31221*	neuropeptides/cholinergic neurons	Croset et al., 2018
3	***VGlut***/*CG34355/CG9650*	Glutamatergic neurons	Croset et al., 2018
4	***SoxN***/*CG9650/*__*klg/scro*__	optic lobe	Davie et al., 2018
			Shimozono et al., 2019
5	***pros/dati/****spab*	Central brain neurons type A	Davie et al., 2018
6	*toy/bi*	undetermined	
7	***pros***/*br/beat-IIIc/*__*dati*__	Central brain neurons type A	Davie et al., 2018
8	*sosie/*__*acj6/*__*nAChRalpha6*__*/ct*__	olfactory projection neurons	Croset et al., 2018
9	*CG14989/*__*Gad1/Lim3*__	GABAergic neurons	Davie et al., 2018
			Croset et al., 2018
10	*mbl/CG31345/*__*Imp/ChaT*__	Central brain type B cholinergic neurons	Davie et al., 2018
			Croset et al., 2018
11	***jdp***/*Pka-R2/*__*Rgk1/rut*__	Kenyon cells	Murakami et al., 2017
			Widmer et al., 2018
12	*CG14989/ct/*__*Gad1*__	GABAergic neurons	Croset et al., 2018
13	***Obp44a***/*CG8369/CG15201/*__*MtnA*__	Glia	DeSalvo et al., 2014
14	*CG18598/pdm3/vvl/*__*scro/jus*__	optic lobe neurons	Davie et al., 2018
			Cosmanescu et al., 2018
15	*Pka-C3/CG2016/sosie*	undetermined	
16	*CNMaR/*__*SoxN*__*/*__*Sox102F*__*/*__*acj6*__	optic lobe and antennal lobe neurons	Croset et al., 2018
			Schilling et al., 2019
17	***Eaat1***/*CG2016/CG15522*	Astrocytes	MacNamee et al., 2016
			Freeman, 2015
			Stacey et al., 2010
18	***DIP-theta***/*CG42458/aop/*__*scro*__	optic lobe neurons	Davie et al., 2018
			Comanescu et al., 2018
19	*CG8369/*__*CG1552*__*/*__*Gs2*__	Glia	Huang et al., 2015
			Strauss et al., 2015
			Mazaud et al., 2019
20	*Pka-R2/crb/*__*Rgk1*__	Kenyon cells	Murakami et al., 2017
21	***VGlut***/*mbl/Proc/*__*Imp*__	Central Brain type 2 glutamatergic neurons	Croset et al., 2018
			Davie et al., 2018
22	***CG3168/***__*Cys*__ → *Cys*/***SPARC***	Surface glia and fat body	DeSalvo et al., 2014
			Shahab et al., 2015
23	*Nos/Octbeta1R*__*/Gad1/vMat*__	GABAergic and monoaminergic neurons	Croset et al., 2018

Cluster	Cluster defining genes	Cluster annotation	References
24	***Vmat***/*Hsp23/CG457784//*	Central brain type 2	
	Imp/***Nep1***	monoaminergic neurons	Croset et al., 2018
			Turrel et al., 2016
			Davie et al., 2018
25	*bi/shakB/Octbeta1R*	undetermined	
26	*CG31345/CG18598/shakB*	undetermined	
27	*CG14687/****Dh31/Nep1/jdp***	mushroom body and ellipsoid body	Ye et al., 2004
			Turrel et al., 2016
			Croset et al., 2018
			Nixon et al., 2019
28	*CG43795/CG14274/Frq1*	undetermined	
29	***DAT/Vmat/ple***	Dopaminergic neurons	Croset et al., 2018
30	***Arr2/Arr1/ninaE***	Photoreceptor cells	FlyBase, Thurmond et al., 2019
31	*CG2016/CG6044/CG42540*	undetermined	
32	*lncRNA:CR45223/kn/sNPF*	undetermined	
33	*CG8034/****VGlut****/Ca-alpha1T*	Glutamatergic neurons	Croset et al., 2018
34	***Tk/Nplp1/nAChRalpha6***	Tachykinin/neuropeptidergic neurons	Ignell et al., 2009
			Asahina et al., 2014
35	*lncRNA:CR45223/****sNPF/Gad1***	Central brain GABAergic neurons	Croset et al., 2018
			Davie et al., 2018

Table S4: Characterization of cell clusters through combinations of biomarkers. Genes indicated in bold font were used to annotate the clusters.

Table S5: Genes that were consistently upregulated and downregulated across multiple clusters due to acute cocaine exposure. Refer to Supplemental_Table_S5.xlsx

Table S6: Results from differential expression analysis for each cluster from the male and female datasets combined. Refer to Supplemental_Table_S6.xlsx

Table S7: Results from differential expression analysis for each cluster from the male dataset. Refer to Supplemental_Table_S7.xlsx

Table S8: Results from differential expression analysis for each cluster from the female dataset. Refer to Supplemental_Table_S8.xlsx

Table S9: Quantification of shared and unique differentially expressed genes due to exposure to cocaine between the males and females within each cluster. Refer to Supplemental_Table_S9.xlsx

Table S10: Functional enrichment analyses of differentially expressed genes in selected clusters due to cocaine exposure. Refer to Supplemental_Table_S10.xlsx

Supplemental Figures

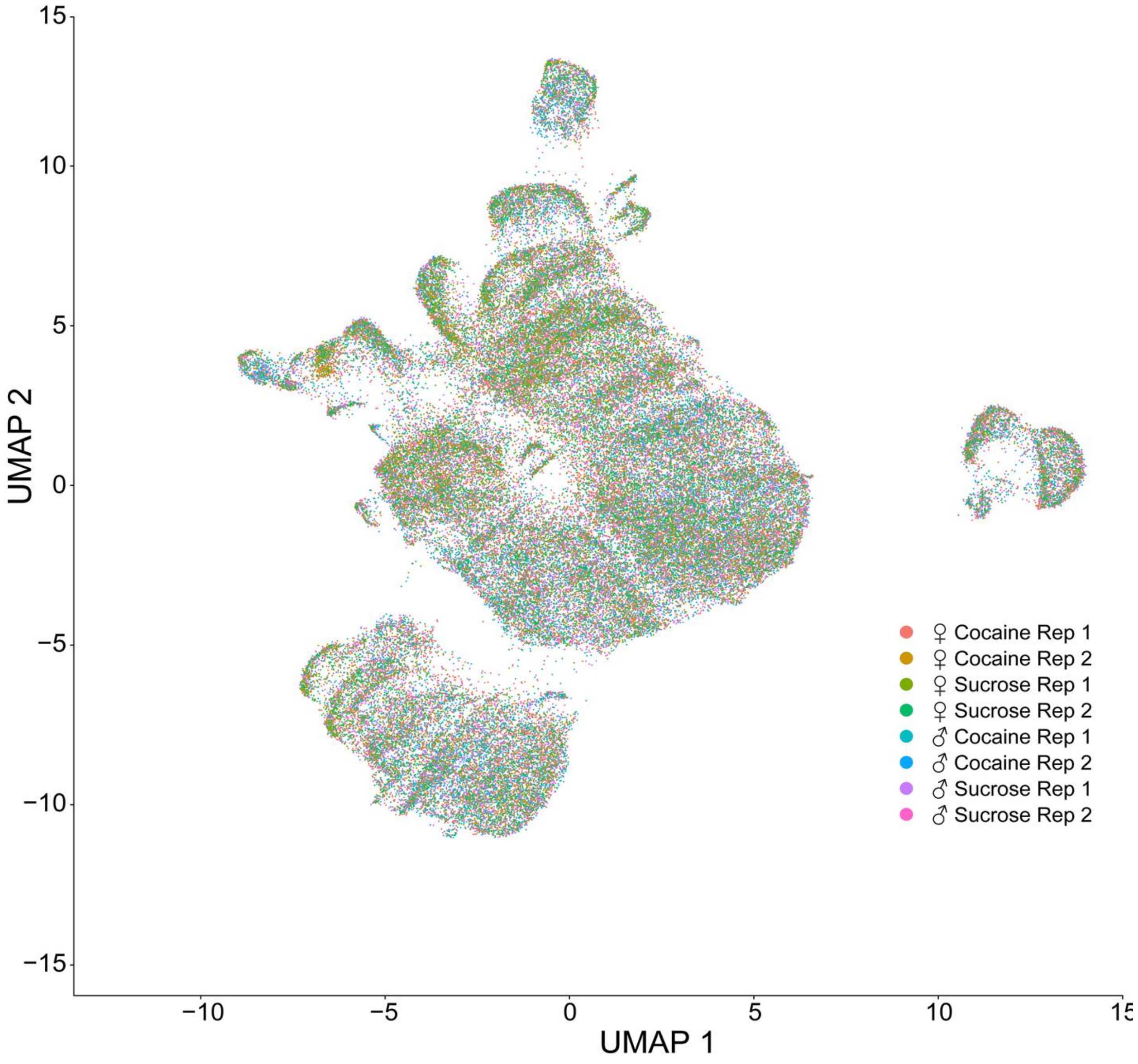

Figure S1: Visualization of gene expression patterns using UMAP projections. Gene expression patterns of single cells (n = 86,224) from all 8 (2 ♀cocaine, 2 ♀sucrose, 2 ♂cocaine, 2 ♂sucrose) samples were visualized in low dimensional space using a graph-based, non-linear dimensionality reduction method (UMAP). Individual dots represent each cell and the colors of the dots represent the samples to which the cells belong.

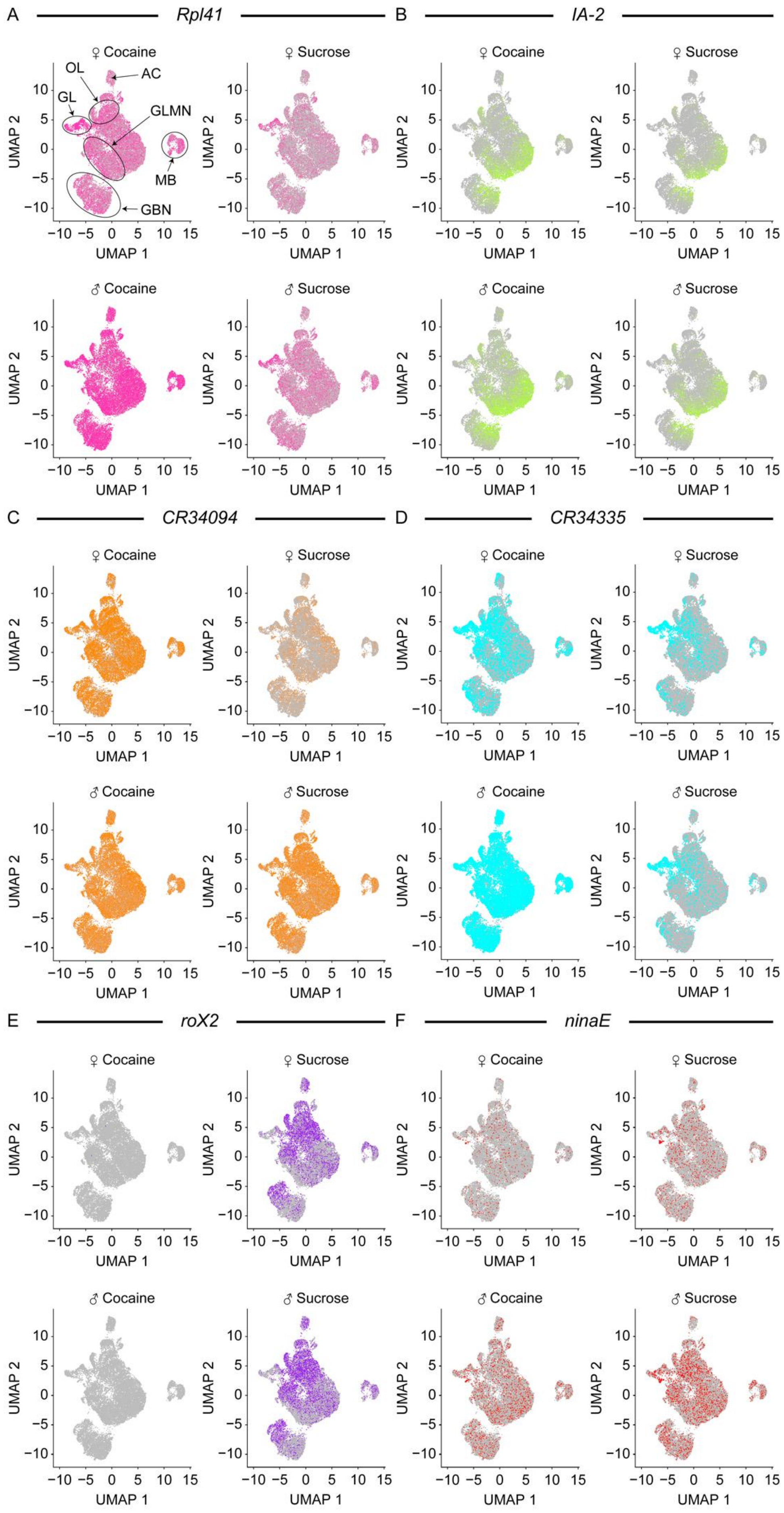
A
Rpl41
B
IA-2
C
CR34094
D
CR34335
E
roX2
F
ninaE
♀ Cocaine
♀ Sucrose
♂ Cocaine
♂ Sucrose
OL
AC
GL
GLMN
MB
GBN
UMAP 1
UMAP 2

Figure S2: UMAP visualization of expression patterns for genes that respond globally to cocaine exposure. Visualization of gene expression patterns was performed using UMAP projections. Each dot represents a cell within the integrated dataset from all 8 samples. The color gradients of the dots represent the normalized and scaled expression value of the genes that respond globally due to acute cocaine exposure in each sample group (separated based on sex and condition). Identities of specific clusters and groups of cell types are indicated in the *Rpl41* panel A: GL - Glia, OL - Optic Lobe, AC - astrocytes, GLMN - Glutamatergic neurons, MB - Mushroom Body, GBN - GABAergic neurons.

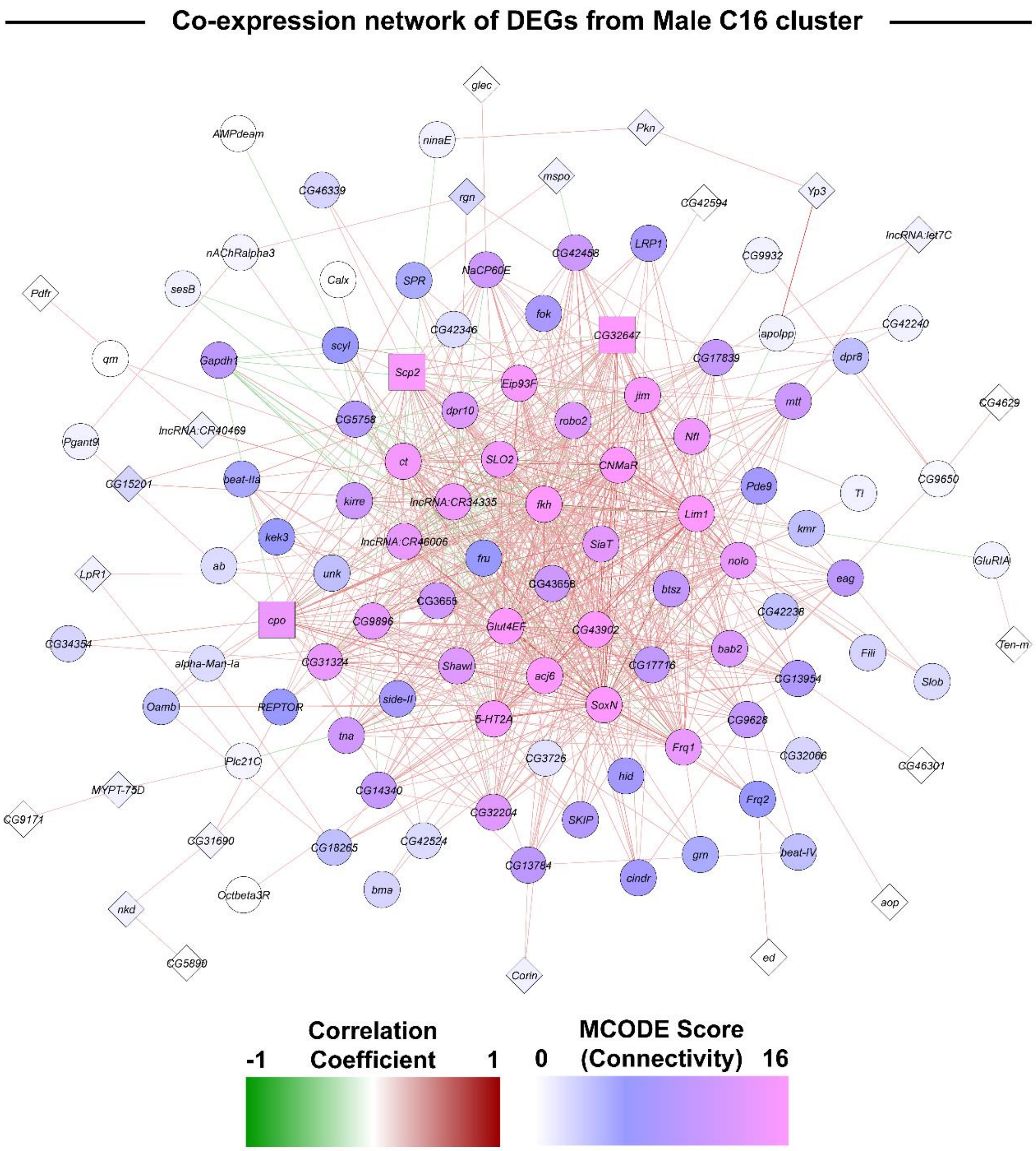

Figure S3: Full co-expression network analysis of DEGs from the male C16 cluster. Network constructed from Pearson Coefficient based co-expression values calculated from scaled data of genes that were differentially expressed (filtered for $|\log_e FC| > 0.5$, Bonferroni adjusted P value < 0.05) due to cocaine exposure in C16 of the male dataset. Co-expressions have been filtered using Random Matrix Theory. The colors of the dots represent the connectivity index derived from MCODE scores. The colors of edges represent the positive (red) and negative (green) correlations.

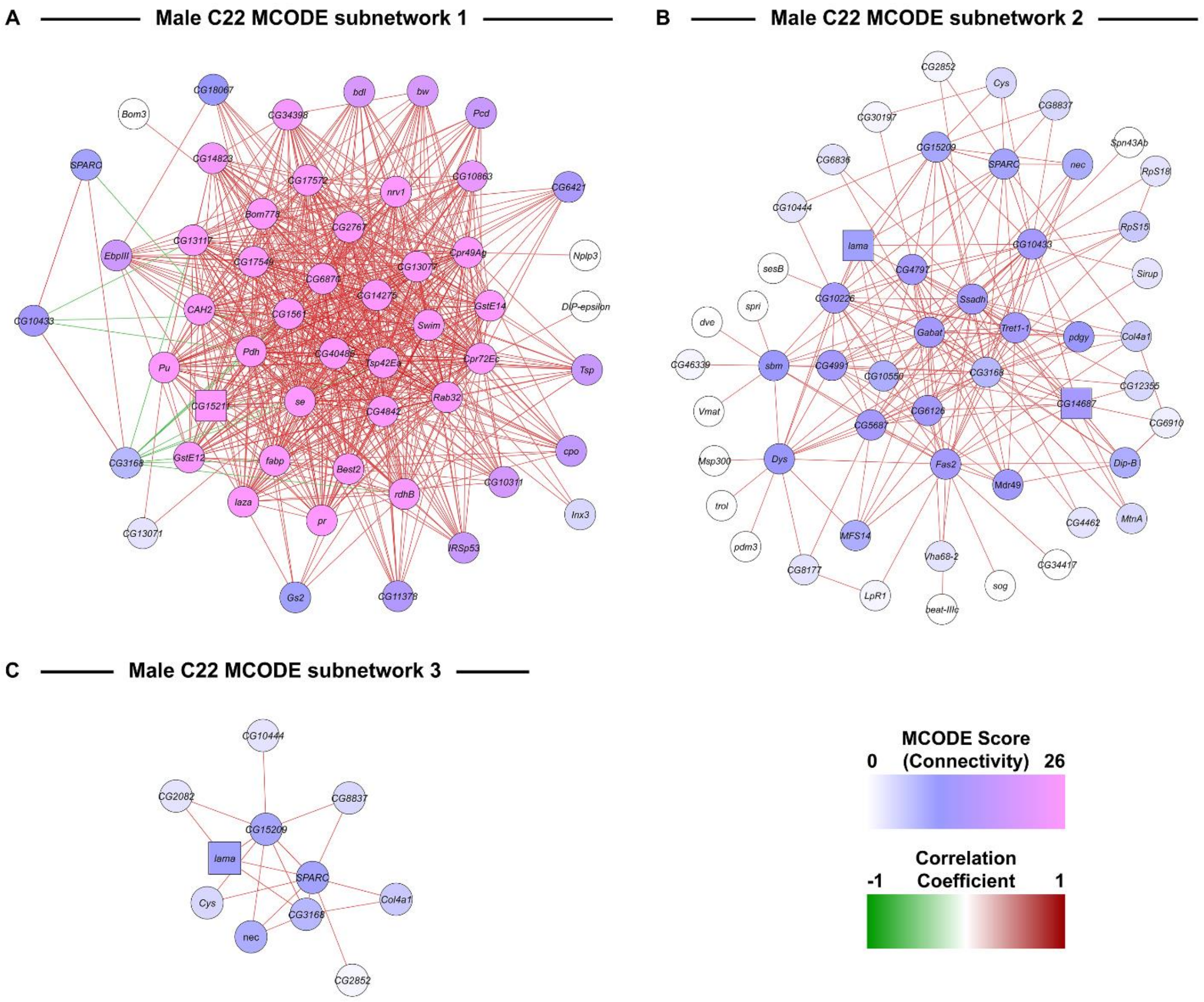

Figure S4: MCODE Subnetworks from co-expression network analysis of DEGs from the male C22 cluster. Network constructed from Pearson Coefficient based co-expression values calculated from scaled data of genes that were differentially expressed (filtered for $|\log_e FC| > 0.5$, Bonferroni adjusted P value < 0.05) due to cocaine exposure in C22 of the male dataset. Co-expression values have been filtered using Random Matrix Theory. (**a-c**) MCODE subnetworks derived from the full network. The colors of the dots represent the connectivity index derived from MCODE scores. The colors of edges represent the positive (red) and negative (green) correlations.

Supplemental Movies

Movie S1: Startle response assay. Refer to Supplemental_Movie_S1.mp4

Movie S2: Grooming behavior of a male exposed to cocaine. Refer to Supplemental_Movie_S2.mp4

Movie S3: Seizure of a male exposed to cocaine. Refer to Supplemental_Movie_S3.mp4

Supplemental References

Asahina K, Watanabe K, Duistermars BJ, Hoopfer E, González CR, Eyjólfsdóttir EA, et al. Tachykinin-expressing neurons control male-specific aggressive arousal in *Drosophila*. *Cell*. 2014;156:221-35.

Cosmanescu F, Katsamba PS, Sergeeva AP, Ahlsen G, Patel SD, Brewer JJ, et al. Neuron-subtype-specific expression, interaction affinities, and specificity determinants of DIP/Dpr cell recognition proteins. *Neuron.* 2018;100:1385-1400.

Croset V, Treiber CD, Waddell S. Cellular diversity in the *Drosophila* midbrain revealed by single-cell transcriptomics. *Elife*. 2018;7 e34550.

Davie K, Janssens J, Koldere D, De Waegeneer M, Pech U, Kreft Ł, et al. A Single-cell transcriptome atlas of the aging *Drosophila* brain. *Cell*. 2018;174:982-98.

DeSalvo MK, Hindle SJ, Rusan ZM, Orng S, Eddison M, Halliwill K, et al. The *Drosophila* surface glia transcriptome: evolutionary conserved blood-brain barrier processes. *Front. Neurosci*. 2014;8:346.

Freeman MR. *Drosophila* central nervous system glia. *Cold Spring Harb. Perspect. Biol*. 2015;7:a020552.

Ignell R, Root CM, Birse RT, Wang JW, Nässel DR, Winther AM. Presynaptic peptidergic modulation of olfactory receptor neurons in *Drosophila*. *Proc. Natl. Acad. Sci. USA*. 2009;106:13070-5.

MacNamee SE, Liu KE, Gerhard S, Tran CT, Fetter RD, Cardona A, et al. Astrocytic glutamate transport regulates a *Drosophila* CNS synapse that lacks astrocyte ensheathment. *J. Comp. Neurol*. 2016;524:1979-98.

Mazaud D, Kottler B, Gonçalves-Pimentel C, Proelss S, Tüchler N, Deneubourg C, et al. Transcriptional regulation of the glutamate/GABA/glutamine cycle in adult glia controls motor activity and seizures in *Drosophila*. *J. Neurosci*. 2019;39:5269-83.

Murakami S, Minami-Ohtsubo M, Nakato R, Shirahige K, Tabata T. Two components of aversive memory in *Drosophila*, anesthesia-sensitive and anesthesia resistant memory, require distinct domains within the Rgk1 small GTPase. *J. Neurosci*.2017;37:5496-510.

Nixon KCJ, Rousseau J, Stone MH, Sarikahya M, Ehresmann S, Mizuno S, et al. A syndromic neurodevelopmental disorder caused by mutations in SMARCD1, a core SWI/SNF subunit needed for context-dependent neuronal gene regulation in flies. *Am. J. Hum. Genet*. 2019;104:596-610.

Schilling T, Ali AH, Leonhardt A, Borst A, Pujol-Martí J. Transcriptional control of morphological properties of direction-selective T4/T5 neurons in *Drosophila*. *Development.* 2019;146: dev169763.

Shahab J, Baratta C, Scuric B, Godt D, Venken KJ, Ringuette MJ. Loss of SPARC dysregulates basal lamina assembly to disrupt larval fat body homeostasis in *Drosophila melanogaster*. *Dev. Dyn*. 2015;244:540-52.

Shimozono M, Osaka J, Kato Y, Araki T, Kawamura H, Takechi H, et al. Cell surface molecule, Klingon, mediates the refinement of synaptic specificity in the *Drosophila* visual system. *Genes Cells*. 2019;24:496-510.

Stacey SM, Muraro NI, Peco E, Labbé A, Thomas GB, Baines RA, et al. *Drosophila* glial glutamate transporter Eaat1 is regulated by fringe-mediated notch signaling and is essential for larval locomotion. *J. Neurosci*. 2010;30:14446-57.

Strauss AL, Kawasaki F, Ordway RW. A distinct perisynaptic glial cell type forms tripartite neuromuscular synapses in the *Drosophila* adult. *PLoS One.* 2015;10:e0129957.

Thurmond J, Goodman JL, Strelets VB, Attrill H, Gramates LS, Marygold SJ, et al. FlyBase 2.0: the next generation. *Nucleic Acids Res*. 2019;47:D759-D765.

Turrel O, Lampin-Saint-Amaux A, Préat T, Goguel V. *Drosophila* neprilysins are involved in middle-term and long-term memory. *J. Neurosci.* 2016;36:9535-46.

Widmer YF, Bilican A, Bruggmann R, Sprecher SG. Regulators of long-term memory revealed by mushroom body-specific gene expression profiling in *Drosophila melanogaster. Genetics*. 2018;209:1167-81.

Ye Y, Xi W, Peng Y, Wang Y, Guo A. Long-term but not short-term blockade of dopamine release in *Drosophila* impairs orientation during flight in a visual attention paradigm. *Eur. J. Neurosci.* 2004;20:1001-7.
